# Supplementary material for: Effects of trust, risk perception, and health behavior on COVID-19 disease burden: Evidence from a multi-state US survey
Source: PLoS One. 2022 May 20;17(5):e0268302. doi: 10.1371/journal.pone.0268302 (PMC9122183; doi:10.1371/journal.pone.0268302)
Supplement: S1 Appendix — (DOCX) [file pone.0268302.s001.docx]

COVID survey for rural states - FINAL

Start of Block: Consent Form

**RESEARCH PARTICIPANT INFORMATION AND CONSENT FORM**
 

**What is the purpose of this study?**
We invite you to take an online survey about the impacts of the coronavirus disease 2019 pandemic (COVID-19) on U.S. residents. The information you provide will help researchers at the University of Idaho and public health officials take action to protect communities from COVID-19. Please take the survey if you are an adult 18 years of age or older. 
 
**What will I do in this study?**
You will fill out a survey that should take about 20 minutes to complete. You may save your responses at any time and return to the survey at a later time/date.  
 
**Are there any risks to me for participating in the research?**  
Participation in this survey is voluntary. There are no foreseen risks to participation. You may stop this survey at any time or skip a question if you are uncomfortable answering it. We will keep your information anonymous and confidential at all times.
 
**How will I be compensated for my participation?**
As a token of appreciation for your participation, please accept the $1 gift we sent you with the invitation letter.    
 
**Who should I contact for questions or concerns?**
If you have any comments, questions, or concerns about the survey, please contact Ben Ridenhour by email: covsurvey@uidaho.edu or phone: 208-885-8607. 
 
**Statement of consent:**
By continuing with this survey I am indicating that I am 18 years of age or older and I consent to participate in this research.

| Page Break |  |
| --- | --- |

End of Block: Consent Form

Start of Block: Risk Perception and Behavioral Responses

The following questions are about the coronavirus disease 2019 pandemic (abbreviated as COVID-19). For the purposes of this survey, we will reference the disease as “COVID-19”. 
Please choose only one answer unless the question says choose all that apply.

| 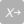 |
| --- |

1
Since March 2020, many states have ordered shutdowns in schools, businesses, work, etc due to COVID-19. To what extent have you limited face-to-face interactions with others outside of your household during these shutdowns?

- By a lot (1)
- Some (2)
- By very little (3)

| 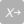 |
| --- |

2 How worried are you about each of the following for getting sick with COVID-19?

|  | Not worried at all (1) | Slightly worried (2) | Moderately worried (3) | Very worried (4) |
| --- | --- | --- | --- | --- |
| Yourself (2_1) |  |  |  |  |
| Your family (2_2) |  |  |  |  |
| Others in your community (2_3) |  |  |  |  |

| 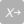 |
| --- |

3 How much of a threat do you believe the COVID-19 pandemic is for each of the following?

|  | Not a threat (1) | Minor threat (2) | Major threat (3) |
| --- | --- | --- | --- |
| Your personal health (3_1) |  |  |  |
| Your personal financial situation (3_2) |  |  |  |
| Your local education system (3_3) |  |  |  |
| The economy of your community (3_4) |  |  |  |
| The health of your community (3_5) |  |  |  |
| The health of the US population as a whole (3_6) |  |  |  |
| The US economy (3_7) |  |  |  |

| 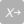 |
| --- |

4 Think about the 12 months PRIOR to when you first heard about COVID-19. How often did you engage in the below activities during that pre-COVID period?

|  | Never (1) | Occasionally (2) | Often (3) | Very often (4) |
| --- | --- | --- | --- | --- |
| Gather indoors with close friends or family who do not live in your home (4_1) |  |  |  |  |
| Gather indoors with a large group of 20-30 friends or co-workers (4_2) |  |  |  |  |
| Eat inside at a restaurant (4_3) |  |  |  |  |
| Attend indoor church or faith services (4_4) |  |  |  |  |
| Go shopping in your town (4_5) |  |  |  |  |
| Go to an appointment for half an hour or longer (e.g. haircut, dental cleaning) (4_6) |  |  |  |  |
| Participate in community activities or events like sports, fairs, and festivals, or let your kids do so (4_7) |  |  |  |  |

| 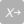 |
| --- |

5 Think about your COVID-19 situation right now. Given this situation, which of the following activities do you engage in currently?

|  | Yes (1) | No (2) |
| --- | --- | --- |
| Gather indoors with close friends or family who do not live in your home (5_1) |  |  |
| Gather indoors with a large group of 20-30 friends or co-workers (5_2) |  |  |
| Eat inside at a restaurant (5_3) |  |  |
| Attend indoor church or faith services (5_4) |  |  |
| Go shopping in your town (5_5) |  |  |
| Go to an appointment for half an hour or longer (e.g. haircut, dental cleaning) (5_6) |  |  |
| Participate in community activities or events like sports, fairs, and festivals, or let your kids do so (5_7) |  |  |
| Wear a face mask in public (5_8) |  |  |

| 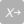 |
| --- |

6
Suppose that new cases of COVID-19 are increasing in your state but no new cases are reported in your community. Which of the following activities would you feel comfortable doing?

|  | Yes (1) | No (2) |
| --- | --- | --- |
| Gather indoors with close friends or family who do not live in your home (6_1) |  |  |
| Gather indoors with a large group of 20-30 friends or co-workers (6_2) |  |  |
| Eat inside at a restaurant (6_3) |  |  |
| Attend indoor church or faith services (6_4) |  |  |
| Go shopping in your town (6_5) |  |  |
| Go to an appointment for half an hour or longer (e.g. haircut, dental cleaning) (6_6) |  |  |
| Participate in community activities or events like sports, fairs, and festivals, or let your kids do so (6_7) |  |  |
| Wear a face mask in public (6_8) |  |  |

| 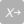 |
| --- |

7 Suppose that new cases of COVID-19 are increasing in your community but no cases were reported among people you are around regularly. Which of the following activities would you feel comfortable doing?

|  | Yes (1) | No (2) |
| --- | --- | --- |
| Gather indoors with close friends or family who do not live in your home (7_1) |  |  |
| Gather indoors with a large group of 20-30 friends or co-workers (7_2) |  |  |
| Eat inside at a restaurant (7_3) |  |  |
| Attend indoor church or faith services (7_4) |  |  |
| Go shopping in your town (7_5) |  |  |
| Go to an appointment for half an hour or longer (e.g. haircut, dental cleaning) (7_6) |  |  |
| Participate in community activities or events like sports, fairs, and festivals, or let your kids do so (7_7) |  |  |
| Wear a face mask in public (7_8) |  |  |

| 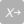 |
| --- |

8 Suppose that new cases of COVID-19 are increasing in your community and there were multiple cases reported among people you are around regularly. Which of the following activities would you feel comfortable doing?

|  | Yes (1) | No (2) |
| --- | --- | --- |
| Gather indoors with close friends or family who do not live in your home (8_1) |  |  |
| Gather indoors with a large group of 20-30 friends or co-workers (8_2) |  |  |
| Eat inside at a restaurant (8_3) |  |  |
| Attend indoor church or faith services (8_4) |  |  |
| Go shopping in your town (8_5) |  |  |
| Go to an appointment for half an hour or longer (e.g. haircut, dental cleaning) (8_6) |  |  |
| Participate in community activities or events like sports, fairs, and festivals, or let your kids do so (8_7) |  |  |
| Wear a face mask in public (8_8) |  |  |

End of Block: Risk Perception and Behavioral Responses

Start of Block: Social influence I

You are about to be asked 3 questions about activities in daily life. To answer these questions, suppose that new cases of COVID-19 are increasing in your state, but there are no new cases in your community.

| 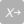 |
| --- |

SI1: 9
Suppose your town has no mask-wearing requirement. You go to a store. Would you wear a mask if...

- No other customer wears a mask (1)
- Some customers (1 in 4) wear a mask (2)
- Half of the customers (2 in 4) wear a mask (3)
- Most customers (3 in 4) wear a mask (4)
- Every customer wears a mask (5)
- I would NOT wear a mask even if all customers wear a mask (6)

| 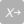 |
| --- |

SI1: 10 Suppose a friend is having an indoor gathering. Would you decline (or say “no”) to an invitation to this gathering if...

- No one else declined (1)
- Some guests (1 in 4) declined (2)
- Half of the guests (2 in 4) declined (3)
- Most guests (3 in 4) declined (4)
- All guests declined (5)
- I would ACCEPT the invitation even if everyone else declined (6)

| 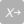 |
| --- |

SI1: 11 Suppose a close relative is having an indoor birthday party of your family and close friends. Would you decline (or say “no”) to an invitation to this party if...

- No one else declined (1)
- Some guests (1 in 4) declined (2)
- Half of the guests (2 in 4) declined (3)
- Most guests (3 in 4) declined (4)
- All guests declined (5)
- I would ACCEPT the invitation even if everyone else declined (6)

End of Block: Social influence I

Start of Block: Social Influence II

You are about to be asked 3 questions about activities in daily life. To answer these questions, suppose that new cases of COVID-19 are increasing in your community, but there are no cases among people you interact with regularly.

| 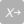 |
| --- |

SI2: 9 Suppose your town has no mask-wearing requirement. You go to a store. Would you wear a mask if...

- No other customer wears a mask (1)
- Some customers (1 in 4) wear a mask (2)
- Half of the customers (2 in 4) wear a mask (3)
- Most customers (3 in 4) wear a mask (4)
- Every customer wears a mask (5)
- I would NOT wear a mask even if all customers wear a mask (6)

| 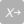 |
| --- |

SI2: 10 Suppose a friend is having an indoor gathering. Would you decline (or say “no”) to an invitation to this gathering if...

- No one else declined (1)
- Some guests (1 in 4) declined (2)
- Half of the guests (2 in 4) declined (3)
- Most guests (3 in 4) declined (4)
- All guests declined (5)
- I would ACCEPT the invitation even if everyone else declined (6)

| 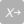 |
| --- |

SI2: 11 Suppose a close relative is having an indoor birthday party of your family and close friends. Would you decline (or say “no”) to an invitation to this party if...

- No one else declined (1)
- Some guests (1 in 4) declined (2)
- Half of the guests (2 in 4) declined (3)
- Most guests (3 in 4) declined (4)
- All guests declined (5)
- I would ACCEPT the invitation even if everyone else declined (6)

End of Block: Social Influence II

Start of Block: Social Influence III

You are about to be asked 3 questions about activities in daily life. To answer these questions, suppose that new cases of COVID-19 are increasing in your community AND there are multiple cases among people you interact with regularly.

| 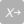 |
| --- |

SI3: 9 Suppose your town has no mask-wearing requirement. You go to a store. Would you wear a mask if...

- No other customer wears a mask (1)
- Some customers (1 in 4) wear a mask (2)
- Half of the customers (2 in 4) wear a mask (3)
- Most customers (3 in 4) wear a mask (4)
- Every customer wears a mask (5)
- I would NOT wear a mask even if all customers wear a mask (6)

| 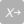 |
| --- |

SI3: 10 Suppose a friend is having an indoor gathering. Would you decline (or say “no”) to an invitation to this gathering if...

- No one else declined (1)
- Some guests (1 in 4) declined (2)
- Half of the guests (2 in 4) declined (3)
- Most guests (3 in 4) declined (4)
- All guests declined (5)
- I would ACCEPT the invitation even if everyone else declined (6)

| 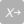 |
| --- |

SI3: 11 Suppose a close relative is having an indoor birthday party of your family and close friends. Would you decline (or say “no”) to an invitation to this party if...

- No one else declined (1)
- Some guests (1 in 4) declined (2)
- Half of the guests (2 in 4) declined (3)
- Most guests (3 in 4) declined (4)
- All guests declined (5)
- I would ACCEPT the invitation even if everyone else declined (6)

End of Block: Social Influence III

Start of Block: Social Influence Cont.

| 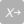 |
| --- |

12 Which one of the below statements best describes your view about COVID-19?

- COVID-19 is not real (1)
- COVID-19 is real but not serious (2)
- COVID-19 is real and serious (3)

| 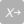 |
| --- |

13 How strongly do you agree or disagree that shutting down schools and businesses to reduce the spread of COVID-19 has come at too great an economic cost?

- Strongly agree (1)
- Somewhat agree (2)
- Somewhat disagree (3)
- Strongly disagree (4)

| 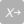 |
| --- |

14 About how common is it for your family and close friends to share similar views as yours on the economic cost of school and business shutdowns due to COVID-19? Please give us your best estimate.

- Very rare (fewer than 1 in 4 people) (1)
- Rare (1 in 4 people) (2)
- About even (2 in 4 people) (3)
- Common (3 in 4 people) (4)
- Very common (more than 3 in 4 people) (5)

| 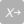 |
| --- |

15 About how common is it for people in your community to share similar views as yours on the economic cost of school and business shutdowns due to COVID-19? Please give us your best estimate.

- Very rare (fewer than 1 in 4 people) (1)
- Rare (1 in 4 people) (2)
- About even (2 in 4 people) (3)
- Common (3 in 4 people) (4)
- Very common (more than 3 in 4 people) (5)

End of Block: Social Influence Cont.

Start of Block: Work and Economy

| 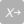 |
| --- |

16 What was your employment status at the beginning of 2020, prior to the COVID-19 pandemic?

- I was employed for wages full time (1)
- I was employed for wages part time (2)
- I was self-employed (3)
- I was unemployed and looking for work (4)
- I was unemployed and not looking for work (e.g. retired, disabled) (5)

| 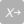 |
| --- |

17 Did you experience any of the following because of the COVID-19 pandemic?

|  | Yes (1) | No (2) |
| --- | --- | --- |
| Worked fewer hours (17_1) |  |  |
| Was laid off (17_2) |  |  |
| Took a pay cut (17_3) |  |  |
| Had to take unpaid time off (17_4) |  |  |
| None of the above (17_5) |  |  |
| Not applicable (e.g. not employed) (17_6) |  |  |

| 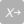 |
| --- |

18 How hard is it for you to pay your household bills right now?

- Not hard (1)
- Somewhat hard (2)
- Very hard (3)

| 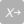 |
| --- |

19 If you were to develop mild symptoms of a respiratory infection this week (e.g. cough or fever or shortness of breath), would you...

|  | Yes (1) | No (2) |
| --- | --- | --- |
| Isolate at home for at least 10 days (19_1) |  |  |
| Isolate at home until the symptoms improve (19_2) |  |  |
| Try to get tested for COVID-19 (19_3) |  |  |
| Go to work (19_4) |  |  |
| Continue seeing friends and family (19_5) |  |  |

Skip To: 20 If If you were to develop mild symptoms of a respiratory infection this week (e.g. cough or fever or... = Go to work [ Yes ]

Skip To: End of Block If If you were to develop mild symptoms of a respiratory infection this week (e.g. cough or fever or... = Go to work [ No ]

| 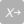 |
| --- |

20 If you were to develop mild symptoms of a respiratory infection this week (e.g. cough or fever or shortness of breath), you would go to work because...

|  | Yes (1) | No (2) |
| --- | --- | --- |
| You are not worried about the coronavirus (20_1) |  |  |
| You need to earn money (20_2) |  |  |
| You are afraid of losing your job or having your hours reduced (20_3) |  |  |
| Your work is too important to miss (20_4) |  |  |
| Not applicable to you (e.g. not employed, work from home) (20_5) |  |  |
| Other reasons (please specify) (20_6) |  |  |

End of Block: Work and Economy

Start of Block: Contact Tracing, Vaccines, Trust

| 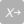 |
| --- |

21 Contact tracing is the idea that when someone tests positive for COVID-19, the people they have come in close contact with should be notified and tested. If a contact tracer called you because you may have been exposed, how likely are you to follow each of the recommendations below?

|  | Very Unlikely (1) | Unlikely (2) | Likely (3) | Very Likely (4) |
| --- | --- | --- | --- | --- |
| Self-isolate at home for 10 days (21_1) |  |  |  |  |
| Get tested for the virus if you develop symptoms (21_2) |  |  |  |  |
| Get tested for the virus even if you do not develop symptoms (21_3) |  |  |  |  |
| Share with the contact tracer the names of people you have been in close contact with recently (21_4) |  |  |  |  |

| 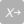 |
| --- |

22 If a vaccine against COVID-19 is approved by the FDA (Food and Drug Administration) and becomes available, would you get vaccinated?

- Yes (1)
- No (2)
- Maybe--it depends on the number of new COVID-19 cases at the time (3)
- Maybe--it depends on the side-effects of the vaccine (4)
- Maybe--it depends on both the number of new COVID-19 cases at the time and the side-effects of the vaccine (5)

| 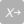 |
| --- |

23 Assuming the vaccine against COVID-19 is highly effective and has side-effects similar to the flu vaccine, when would you get vaccinated? 

- I would get vaccinated no matter what (1)
- If new cases of COVID-19 are steady in my state (not going up or down), but there are no new cases in my community (2)
- If new cases of COVID-19 are increasing rapidly in my state, but there are no new cases in my community (3)
- If new cases of COVID-19 are increasing rapidly in my community, but there are no cases among people I interact with regularly (4)
- If new cases of COVID-19 are increasing rapidly in my community, and there are multiple cases among people I interact with regularly (5)
- I would never get vaccinated (6)

| 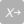 |
| --- |

24 Indicate how informed you feel about the following.

|  | Very informed (1) | Somewhat informed (2) | Not at all informed (3) |
| --- | --- | --- | --- |
| Symptoms of COVID-19 (24_1) |  |  |  |
| How to avoid getting COVID-19 (24_2) |  |  |  |
| How to get tested for COVID-19 (24_3) |  |  |  |
| Steps to take if someone in your household gets COVID-19 (24_4) |  |  |  |

| 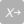 |
| --- |

25 Which of the below statements best reflect the status of COVID-19 in your county right now?

- There have been no known new cases of COVID-19 in my county in the past week (1)
- New cases of COVID-19 have gone down in my county in the past week (2)
- New cases of COVID-19 are steady in my county (not going up or down) in the past week (3)
- New cases of COVID-19 have been increasing in my county in the past week (4)
- I don't know (5)

| 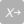 |
| --- |

26 How much do you trust the following individuals/groups to provide you with accurate information about COVID-19?

|  | Not at all (1) | A little bit (2) | Some (3) | A lot (4) |
| --- | --- | --- | --- | --- |
| Governor of your state (26_1) |  |  |  |  |
| State/local public health officials (26_2) |  |  |  |  |
| Mayor or county commissioner (26_3) |  |  |  |  |
| Faith leaders (26_4) |  |  |  |  |
| A doctor or health care provider (26_5) |  |  |  |  |
| The Centers for Disease Control and Prevention (CDC) (26_6) |  |  |  |  |
| The World Health Organization (WHO) (26_7) |  |  |  |  |
| University researchers (e.g. epidemiologists) (26_8) |  |  |  |  |
| The Trump Administration (26_9) |  |  |  |  |

| 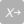 |
| --- |

27 How much information do you get from the following sources?

|  | None (1) | A little bit (2) | Some (3) | A lot (4) |
| --- | --- | --- | --- | --- |
| Mainstream Media (Associated Press, New York Times, Wall Street Journal, USA Today, or Washington Post) (27_1) |  |  |  |  |
| Conservative Media (Fox News, Rush Limbaugh, Breitbart News, One America News, or The Drudge Report) (27_2) |  |  |  |  |
| Mainstream Broadcast Media (ABC News, CBS News, NBC News, Univision) (27_3) |  |  |  |  |
| Liberal Media (MSNBC, Vox, Vice, or Huffington Post) (27_4) |  |  |  |  |
| Online News Aggregators (Google News or Yahoo News) (27_5) |  |  |  |  |
| Social Media (Facebook, Twitter, WhatsApp, or YouTube) (27_6) |  |  |  |  |
| The radio (27_7) |  |  |  |  |

End of Block: Contact Tracing, Vaccines, Trust

Start of Block: School; Health

| 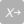 |
| --- |

28 Do you have any children under 18 living with you who attend school outside of your house?

- Yes (1)
- No (2)

Skip To: 30 If Do you have any children under 18 living with you who attend school outside of your house? = No

| 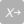 |
| --- |

29 Assuming in-person schooling does not close in your community, when would you keep your children home from school?

If new cases of COVID-19 are steady in my state (not going up or down), but there are no new cases in my community (1)

If new cases of COVID-19 are increasing rapidly in my state, but there are no new cases in my community (2)

If new cases of COVID-19 are increasing rapidly in my community, but there are no cases among people I interact with regularly (3)

If new cases of COVID-19 are increasing rapidly in my community, and there are multiple cases among people I interact with regularly (4)

Regardless of the COVID-19 situation, I would send kids to school as long as school is open (5)

| 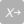 |
| --- |

30 Do you have health insurance?

- Yes (1)
- No (2)

| 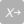 |
| --- |

31
The CDC defines the following categories as high-risk categories for COVID-19. Indicate if any of these apply to you. Choose all that apply.

- I am 65 years or older (1)
- I am immune compromised or am taking medication that suppresses my immune system (2)
- I have a health condition (such as cancer, kidney disease, Type 2 diabetes, heart disease, lung disease, obesity, sickle cell disease) (3)
- I am not in a high-risk category (4)

| 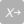 |
| --- |

32
Have you had COVID-19?

- Yes--I tested positive (1)
- Probably--For example, I had symptoms but was never tested (2)
- No--I don't think so (3)
- I don't know or I'm not sure (4)
- I prefer not to say (5)

| 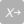 |
| --- |

33 How many people you personally know have had COVID-19 or believe they have had it?

- None (1)
- 1 to 5 (2)
- More than 5 (3)
- I don't know or I'm not sure (4)
- I prefer not to say (5)

End of Block: School; Health

Start of Block: Demographics

| 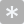 |
| --- |

34 How many people currently live in your household, including you? (Write a number)

________________________________________________________________

| 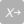 |
| --- |

35 Besides yourself and your spouse/partner, do any people 65 or older currently live in your household?

- Yes (1)
- No (2)

| 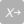 |
| --- |

36 Besides yourself, is there anybody in your household that may be at high risk of severe COVID-19?

- Yes (1)
- No (2)
- I prefer not to say (3)

| 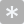 |
| --- |

37 In what year were you born? (Write a number)

________________________________________________________________

| 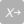 |
| --- |

38 Are you Hispanic or Latino?

- Yes (1)
- No (2)

| 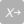 |
| --- |

39 Which categories describe you? Choose all that apply.

- American Indian or Alaska Native (1)
- Asian or Asian-American (2)
- Black or African American (3)
- Native Hawaiian or Other Pacific Islander (4)
- White (5)
- Other (please specify) (6) ________________________________________________

| 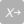 |
| --- |

40 What is the highest level of formal education that you have completed?

- Did not graduate high school (1)
- High school diploma or equivalent (GED) (2)
- Some college (3)
- Associate’s Degree (4)
- College graduate or higher (5)

41
What is your occupation?

________________________________________________________________

| 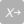 |
| --- |

42 What is your religion/faith?

- Evangelical / Nondenominational Christian (1)
- Catholic (2)
- Mainline Protestant Christian (3)
- Latter-Day Saints (4)
- Jewish (5)
- Islam (6)
- Buddhist (7)
- Atheist (8)
- Agnostic (9)
- Other faiths / other religion (please specify) (10) ________________________________________________

| 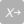 |
| --- |

43 How would you describe your political views?

- Very liberal (1)
- Liberal (2)
- Moderate (3)
- Conservative (4)
- Very conservative (5)
- Libertarian (6)
- Non-political (7)
- Other (please specify) (8) ________________________________________________

| 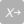 |
| --- |

44 What is your marital status?

- Single, never married (1)
- Married or domestic partnership (2)
- Widowed (3)
- Divorced (4)
- Separated (5)

| 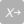 |
| --- |

45 In 2019, what was your total household income from all sources before taxes?

- Less than $25,000 (1)
- $25,000 - 49,999 (2)
- $50,000 - 74,999 (3)
- $75,000 or more (4)

| 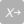 |
| --- |

46 What is your gender?

- Male (1)
- Female (2)
- Do not identify as male or female (3)

| 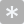 |
| --- |

47 What is your zip code?

________________________________________________________________

End of Block: Demographics

Start of Block: Final Comments

48
Thank you for taking the time to fill out our survey! We really appreciate your participation. If you would like to take part in a follow-up interview about your opinions on COVID-19 please provide us with your contact information (phone number or email) below.

________________________________________________________________

49 If you have any additional comments about COVID-19 or this survey, please feel free write them in the space provided below.

________________________________________________________________

________________________________________________________________

________________________________________________________________

________________________________________________________________

________________________________________________________________

End of Block: Final Comments
