# Supplementary material for: Effects of trust, risk perception, and health behavior on COVID-19 disease burden: Evidence from a multi-state US survey
Source: PLoS One. 2022 May 20;17(5):e0268302. doi: 10.1371/journal.pone.0268302 (PMC9122183; doi:10.1371/journal.pone.0268302)
Supplement: S3 Appendix — (PDF) [file pone.0268302.s003.pdf]

Supplemental Appendix

Contents

Supplemental Appendix Overview . . . 3
Table S3.1: Survey Demographics . . . 5
Table S3.2: RUCA Code Descriptions . . . 7
Figure S3.1: Conceptual Models . . . 8
Figure S3.2: Behavioral Intention Latent Variable Input . . . 9
Figure S3.3: Trust, Health Risk Perception, and Economic Risk Perception Latent Variable Input . . . 10
Table S3.3: Model 1A - Trust to Risk Perception on Disease Burden Latent Output Table . . . 11
Table S3.4: Model 1A - Trust to Risk Perception on Disease Burden Summary Output Table . . . 13
Figure S3.4: Model 1A - Trust to Risk Perception on Disease Burden Full Diagram 14
Figure S3.5: Model 1A - Trust to Risk Perception on Disease Burden Simplified Diagram . . . 15
Table S3.5: Model 2A Risk Perception to Trust on Disease Burden Latent Table Output . . . 16
Table S3.6: Model 2A Risk Perception to Trust on Disease Burden Summary Table Output . . . 18
Figure S3.6: Model 2A - Risk Perception to Trust on Disease Burden Full Diagram 19
Figure S3.7: Model 2A - Risk Perception to Trust on Disease Burden Simplified Diagram . . . 20
Table S3.7: Model 3A Trust and Risk Perception on Disease Burden Latent Table Output . . . 21
Table S3.8: Model 3A Trust and Risk Perception on Disease Burden Summary Table Output . . . 23
Figure S3.8: Model 3A - Trust and Risk Perception on Disease Burden Full Diagram 24

Figure S3.9: Model 3A - Trust and Risk Perception on Disease Burden Simplified Diagram . . . 25
Table S3.9: Model 1B - Trust to Risk Perception on Behavioral Intention Latent Table Output . . . 26
Table S3.10: Model 1B - Trust to Risk Perception on Behavioral Intention Summary Table Output . . . 28
Figure S3.10: Model 1B - Trust to Risk Perception on Behavioral Intention Full Diagram . . . 29
Figure S3.11: Model 1B - Trust to Risk Perception on Behavioral Intention Simplified Diagram . . . 30
Table S3.11: Model 2B - Risk Perception to Trust on Behavioral Intention Latent Table Ouput . . . 31
Table S3.12: Model 2B - Risk Perception to Trust on Behavioral Intention Summary Table Ouput . . . 33
Figure S3.14: Model 2B - Risk Perception to Trust on Behavioral Intention Full Diagram . . . 34
Figure S3.15: Model 2B - Risk Perception to Trust on Behavioral Intention Simplified Diagram . . . 35
Table S3.13: Model 3B - Trust and Risk Perception on Behavioral Intention Latent Table Output . . . 36
Table S3.14: Model 3B - Trust and Risk Perception on Behavioral Intention Summary Table Output . . . 38
Figure S3.12: Model 3B - Trust and Risk Perception on Behavioral Intention Full Diagram . . . 39
Figure S3.13: Model 3B - Trust and Risk Perception on Behavioral Intention Simplified Diagram . . . 40
Table S3.15: Comparing Model Set A . . . 41
Table S3.16: Comparing Model Set B . . . 42

Supplemental Appendix Overview

This supplemental appendix provides full analysis outputs, associated reproducible code, and supportive data. As noted in the associated paper, our analysis describes relationships between social cognitive measures (collected from our COVID-19 survey, conducted in the Fall of 2020) and COVID-19 cases (aggregated by the New York Times - http://github.com/nytimes/covid19).

Data Collection and Transformation

Our structural equation models rely upon the following datasets:

1. COVID-19 daily case data, at a county level. We use aggregate case data that is assembled by the New York Times, and is exposed via their github.com repository (http://github.com/nytimes/covid19). This dataset is within our /data folder, named "COVID_data.csv".

2. COVID-19 survey data. We conducted a COVID-19 health perceptions survey in the Fall of 2020 (n = 1035). This de-identified survey dataset is within our /data folder, named "COVID_survey.csv".

From a transformation perspective, several additional fields are generated:

1. Data is reduced to only cases for Idaho, Texas and Vermont;
2. COVID-19 cases per county are grouped based on 1) cumulative cases between June 1, 2020 and April 30th, 2021, as well as 2) cumulative cases 15 days before and after survey completion.
3. In order to assign rurality, survey responses were grouped into urban and rural classifications, based on the United States Department of Agriculture's Rural/Urban Commuting Areas (RUCA)

Conceptual Models Overview

As note in our associated paper, we evaluated 6 (six) models. Please note the following variable naming conventions: 1) 'Health Risk' and 'Economic Risk' refer to health risk perception and economic risk perception, respectfully. 2) "Behavior" refers to both current behavior (at the time of survey completion) as well as behavioral intention. Shorter terms are used for brevity in diagram notation.

Model Set A: Behavioral intentions as an influence on disease burden. Here we consider the disease burden as cumulative cases across a large window of time (June 2020 to April 2021) to capture a complete view of disease impact.

- Model 1A: Trust effects on economic risk perception and health risk perception, to behavioral intention, and then to disease burden.
- Model 2A: Economic risk perception and health risk perception effects on trust, which impact behavioral intention, and then disease burden.
- Model 3A: Trust, economic risk perception and health risk perception - all impacting behavioral intention directly, and then to disease burden.

Model Set B: Disease burden as an influence on behavioral intention. Here we consider a snapshot of the cases in the two weeks prior to the survey response as a measure of the local disease burden and consider its impact on the expressed behavioral intention.

- Model 1B: Trust effects on economic risk perception and health risk perception, and then behavioral intention.
- Model 2B: Economic risk perception and health risk perception effects on trust, which impact behavioral intention.
- Model 3B: Trust, economic risk perception and health risk perception - all impacting behavioral intention directly.

Table S3.1: Survey Demographics

	ID Pct	TX Pct	VT Pct	Total Pct
**Age**				
Under 25	3.0	4.4	3.4	3.3
25-35	10.9	12.6	11.7	11.4
35-45	15.1	17.0	11.1	13.9
45-55	15.1	16.3	13.4	14.6
55-65	20.6	19.3	25.1	22.1
65-75	25.7	17.8	26.8	25.0
75+	9.6	12.6	8.5	9.6
**Educational Attainment**				
Did not graduate HS	0.4	0.7	0.3	0.4
HS diploma or equivalent	7.5	13.9	7.5	8.4
Some college	19.6	12.4	11.7	15.7
Associate's degree	11.7	9.5	5.3	9.1
College graduate or higher	60.9	63.5	75.1	66.4
**Ethnicity**				
Hispanic	4.3	11.1	0.6	3.9
Non-Hispanic	95.7	88.9	99.4	96.1
**Gender**				
Male	42.0	48.5	45.7	44.2
Female	57.4	50.7	53.8	55.2
Not Identified as Male or Female	0.6	0.7	0.6	0.6
**Income Bracket**				
<$25,000	7.0	8.7	6.7	7.1
$25,000-$49,999	21.1	15.7	17.0	18.9
$50,000-$74,999	24.8	19.7	20.2	22.4
>$74,999	47.2	55.9	56.0	51.6
**Politics**				
Moderate	28.8	35.6	28.5	29.6
Liberal	19.0	15.6	46.6	28.5
Conservative	37.3	34.1	13.3	28.2
Libertarian	4.1	4.4	2.5	3.6
Nonpolitical	8.6	7.4	7.1	7.9
Other	2.2	3.0	2.0	2.2
**Race**				
No Answer	8.0	9.0	3.8	6.7
American Indian or Alaska Native	0.2	0.7	0.0	0.2
Multi-race	3.8	3.5	0.8	2.7
Asian or Asian-American	1.1	4.2	1.1	1.5
Black or African-American	0.2	6.9	0.8	1.4
Native Hawaiian or Other Pacific Islander	0.4	0.0	0.0	0.2
White	83.5	72.9	91.8	84.9

(continued)

	ID Pct	TX Pct	VT Pct	Total Pct
Other	2.9	2.8	1.6	2.4
**Relationship Status**				
Single, never married	8.4	16.3	16.5	12.4
Married or domestic partnership	72.0	67.4	61.7	67.7
Widowed	6.3	7.4	5.3	6.1
Divorced	12.4	6.7	15.1	12.6
Separated	0.8	2.2	1.4	1.2
**Religion**				
Evangelical	19.5	29.1	7.7	16.6
Catholic	12.5	21.6	19.8	16.4
Mainline Protestant	12.7	20.9	16.3	15.2
Latter-Day Saints	21.4	0.0	1.8	11.3
Jewish	0.4	0.0	3.8	1.6
Islam	0.0	2.2	0.3	0.4
Buddhist	1.5	0.0	2.4	1.6
Atheist	6.4	3.7	18.3	10.3
Agnostic	12.7	9.0	16.9	13.7
Other	12.7	13.4	12.7	12.8
**Rurality**				
Rural	36.3	27.8	55.6	41.9
Urban	63.7	72.2	44.4	58.1

Note:

Totals: ID: n = 526 TX: n = 143 VT: n = 365

Age: chi sq. = 12.48, df = 12, p-value = 0.408

Educational Attainment: chi sq. = 31.37, df = 8, p-value = 0

Ethnicity: chi sq. = 29.45, df = 2, p-value = 0

Gender: chi sq. = 2.4, df = 4, p-value = 0.663

Income Bracket: chi sq. = 8.77, df = 6, p-value = 0.187

Politics: chi sq. = 113.04, df = 10, p-value = 0

Race: chi sq. = 71.64, df = 14, p-value = 0

Relationship Status: chi sq. = 24.3, df = 8, p-value = 0.002

Religion: chi sq. = 201.83, df = 18, p-value = 0

Rurality: chi sq. = 46.74, df = 2, p-value = 0

Table S3.2: RUCA Code Descriptions

Code	Description
1	Metropolitan area core: primary flow within an urbanized area (UA)
2	Metropolitan area high commuting: primary flow 30% or more to a UA
3	Metropolitan area low commuting: primary flow 10% to 30% to a UA
4	Micropolitan area core: primary flow within an Urban Cluster of 10,000 to 49,999 (large UC)
5	Micropolitan high commuting: primary flow 30% or more to a large UC
6	Micropolitan low commuting: primary flow 10% to 30% to a large UC
7	Small town core: primary flow within an Urban Cluster of 2,500 to 9,999 (small UC)
8	Small town high commuting: primary flow 30% or more to a small UC
9	Small town low commuting: primary flow 10% to 30% to a small UC
10	Rural areas: primary flow to a tract outside a UA or UC
99	Not coded: Census tract has zero population and no rural-urban identifier information

Figure S3.1: Conceptual Models

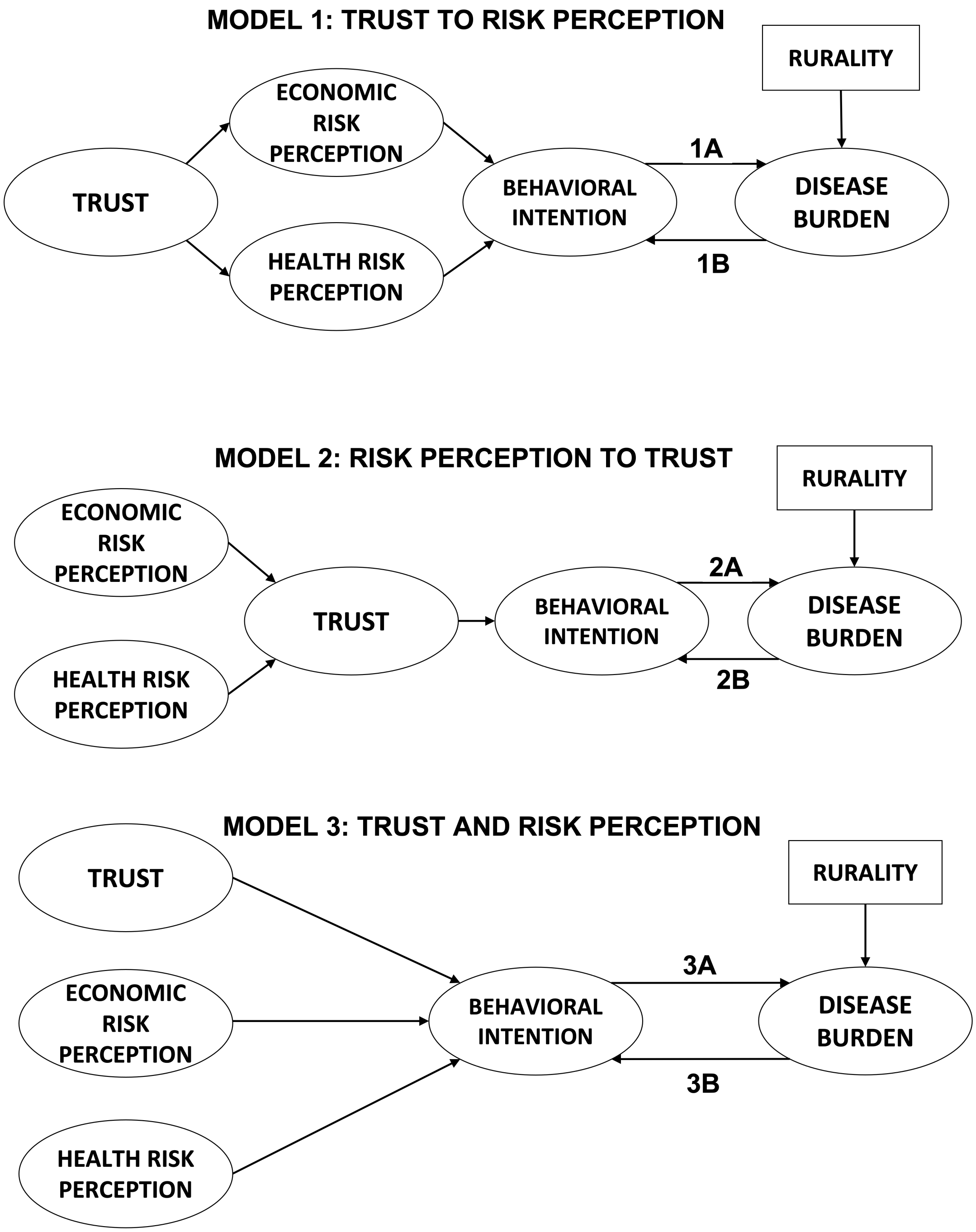

Figure S3.2: Behavioral Intention Latent Variable Input

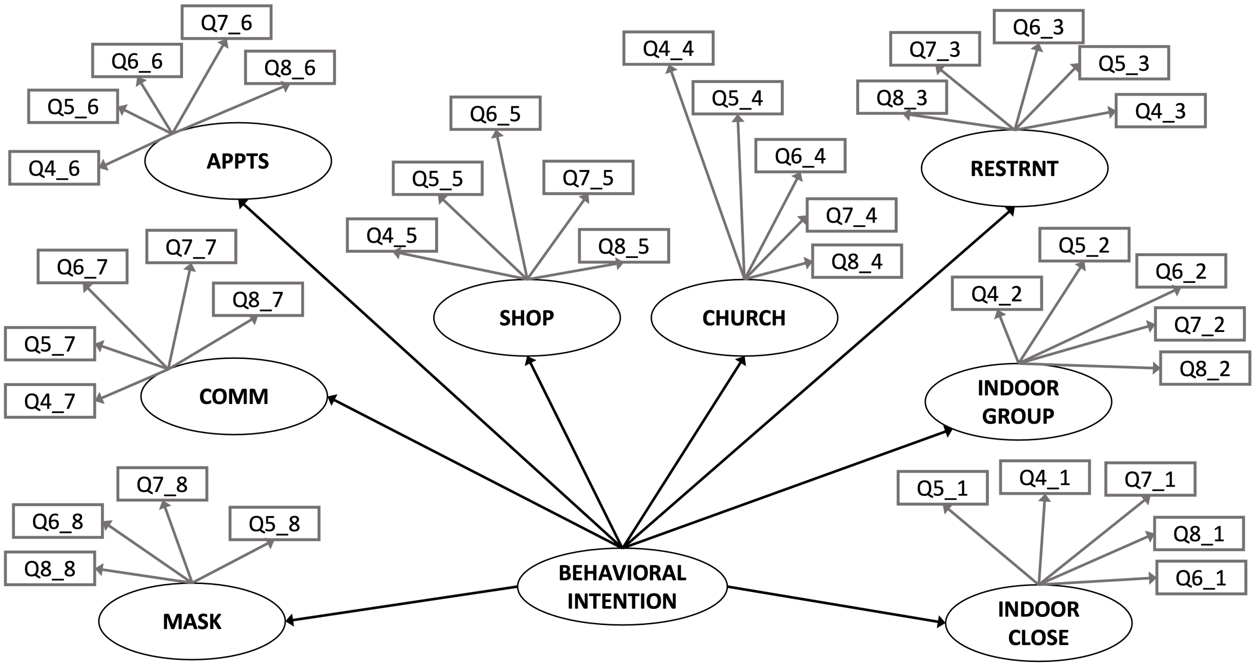

Figure S3.3: Trust, Health Risk Perception, and Economic Risk Perception Latent Variable Input

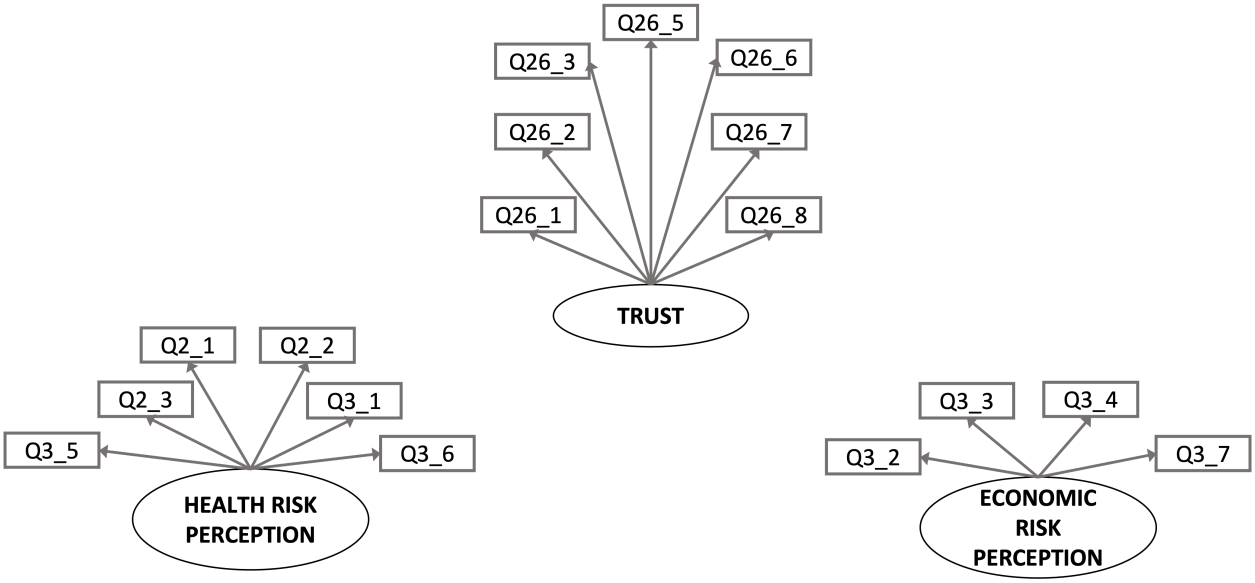

Table S3.3: Model 1A - Trust to Risk Perception on Disease Burden Latent Output Table

Latent Variable	Regression Variable	std.est	std.error	z	p-value
trust	Q26_1	0.4402378	0.000	NA	NA
	Q26_2	0.6957996	0.133	11.896	0.000
	Q26_3	0.5373508	0.115	10.579	0.000
	Q26_5	0.6372721	0.126	11.474	0.000
	Q26_6	0.7719076	0.142	12.352	0.000
	Q26_7	0.8352050	0.150	12.661	0.000
	Q26_8	0.7940041	0.145	12.467	0.000
health_risk	Q2_1	0.8186911	0.000	NA	NA
	Q2_2	0.8111150	0.037	26.906	0.000
	Q2_3	0.7461957	0.038	23.981	0.000
	Q3_1	0.7849239	0.037	25.697	0.000
	Q3_5	0.7302060	0.038	23.298	0.000
	Q3_6	0.7582916	0.038	24.508	0.000
econ_risk	Q3_2	0.3831582	0.000	NA	NA
	Q3_3	0.5472033	0.167	8.539	0.000
	Q3_4	0.8019584	0.233	8.978	0.000
	Q3_7	0.5660691	0.171	8.645	0.000
indoor_close	Q4_1	0.0966251	0.000	NA	NA
	Q5_1	-0.7882445	3.108	-2.628	0.009
	Q6_1	-0.8734829	3.441	-2.631	0.009
	Q7_1	-0.8323181	3.280	-2.630	0.009
	Q8_1	-0.5132929	2.043	-2.602	0.009
indoor_group	Q4_2	0.2115688	0.000	NA	NA
	Q5_2	-0.6257643	0.512	-5.785	0.000
	Q6_2	-0.8831002	0.703	-5.950	0.000
	Q7_2	-0.9099413	0.724	-5.959	0.000
	Q8_2	-0.7522952	0.605	-5.886	0.000
restaurant	Q4_3	0.1353512	0.000	NA	NA
	Q5_3	-0.7021793	1.397	-3.719	0.000
	Q6_3	-0.8135599	1.612	-3.737	0.000
	Q7_3	-0.8934805	1.768	-3.745	0.000
	Q8_3	-0.7611842	1.511	-3.730	0.000
church	Q4_4	0.5079736	0.000	NA	NA
	Q5_4	-0.7313091	0.101	-14.328	0.000
	Q6_4	-0.8819760	0.112	-15.571	0.000
	Q7_4	-0.9203337	0.115	-15.806	0.000
	Q8_4	-0.7999958	0.105	-14.948	0.000
shop	Q4_5	0.0848731	0.000	NA	NA
	Q5_5	-0.5489190	2.894	-2.236	0.025
	Q6_5	-0.7228594	3.793	-2.247	0.025
	Q7_5	-0.8665554	4.542	-2.250	0.024
	Q8_5	-0.6333375	3.329	-2.243	0.025
appointments	Q4_6	0.0903564	0.000	NA	NA
	Q5_6	-0.5914166	2.731	-2.399	0.016
	Q6_6	-0.7474537	3.438	-2.409	0.016
	Q7_6	-0.8606776	3.955	-2.412	0.016
	Q8_6	-0.6483166	2.988	-2.403	0.016
community	Q4_7	0.1400420	0.000	NA	NA
	Q5_7	-0.6494968	1.198	-3.878	0.000

(continued)

Latent Variable	Regression Variable	std.est	std.error	z	p-value
	Q6_7	-0.8692665	1.589	-3.918	0.000
	Q7_7	-0.9287454	1.696	-3.923	0.000
	Q8_7	-0.7815669	1.432	-3.906	0.000
mask	Q5_8	0.8304575	0.000	NA	NA
	Q6_8	0.9371871	0.032	35.612	0.000
	Q7_8	0.9307532	0.032	35.259	0.000
	Q8_8	0.7496445	0.036	25.111	0.000

Table S3.4: Model 1A - Trust to Risk Perception on Disease Burden Summary Output Table

Dependent Variables	Regression Variable	std.est	std.error	z	p-value
trust	ruralbool	-0.0523744	0.027	-1.707	0.088
	femalebool	0.0471461	0.027	1.529	0.126
	whitebool	0.1198891	0.047	3.747	0.000
	age65plusbool	0.0355694	0.029	1.153	0.249
	polVecLiberal	0.1269436	0.036	3.469	0.001
	polVecConservative	-0.4791799	0.049	-9.551	0.000
	polVecLibertarian	-0.2718832	0.087	-7.265	0.000
	polVecNonpolitical	-0.1254426	0.058	-3.671	0.000
	polVecOther	-0.0278693	0.099	-0.899	0.369
health_risk	trust	0.6029439	0.112	9.992	0.000
	ruralbool	0.0176077	0.046	0.630	0.529
	femalebool	0.1073648	0.046	3.804	0.000
	whitebool	-0.1224351	0.078	-4.271	0.000
	age65plusbool	0.2027356	0.050	7.098	0.000
	polVecLiberal	0.0814432	0.060	2.482	0.013
	polVecConservative	-0.0484865	0.068	-1.293	0.196
	polVecLibertarian	-0.0595328	0.133	-1.933	0.053
	polVecNonpolitical	0.0277186	0.096	0.909	0.363
	polVecOther	0.0430336	0.169	1.518	0.129
econ_risk	trust	0.2311012	0.052	3.845	0.000
	ruralbool	-0.0654408	0.030	-1.667	0.096
	femalebool	0.0838719	0.031	2.105	0.035
	whitebool	-0.0647868	0.051	-1.617	0.106
	age65plusbool	0.0037188	0.032	0.095	0.924
	polVecLiberal	0.0506782	0.039	1.109	0.267
	polVecConservative	-0.1508310	0.046	-2.796	0.005
	polVecLibertarian	0.0995256	0.088	2.276	0.023
	polVecNonpolitical	-0.0077235	0.062	-0.183	0.855
	polVecOther	0.0212453	0.109	0.540	0.589
beh_int	econ_risk	0.0577769	0.006	1.573	0.116
	health_risk	-0.7020007	0.020	-2.596	0.009
	ruralbool	0.0063131	0.003	0.268	0.788
	femalebool	0.0747554	0.005	2.007	0.045
	whitebool	-0.0367731	0.006	-1.339	0.180
	age65plusbool	-0.0095526	0.003	-0.386	0.699
	polVecLiberal	-0.0947068	0.006	-2.083	0.037
	polVecConservative	0.2347362	0.013	2.484	0.013
	polVecLibertarian	0.0597318	0.011	1.759	0.079
	polVecNonpolitical	0.0256736	0.006	0.949	0.343
	polVecOther	0.0098239	0.011	0.410	0.682
cumcasesper100	beh_int	0.2637917	1.743	2.482	0.013
	ruralbool	-0.2728009	0.065	-8.442	0.000
Trust		-0.1116543	0.042	-6.093	0.000
Health_risk		-0.1851820	0.031	-7.374	0.000
Rural_via_trust		0.0058478	0.007	1.668	0.095

Note:

chisq = 10470.078 aic = 98774.041 rmsea = 0.071

Figure S3.4: Model 1A - Trust to Risk Perception on Disease Burden Full Diagram

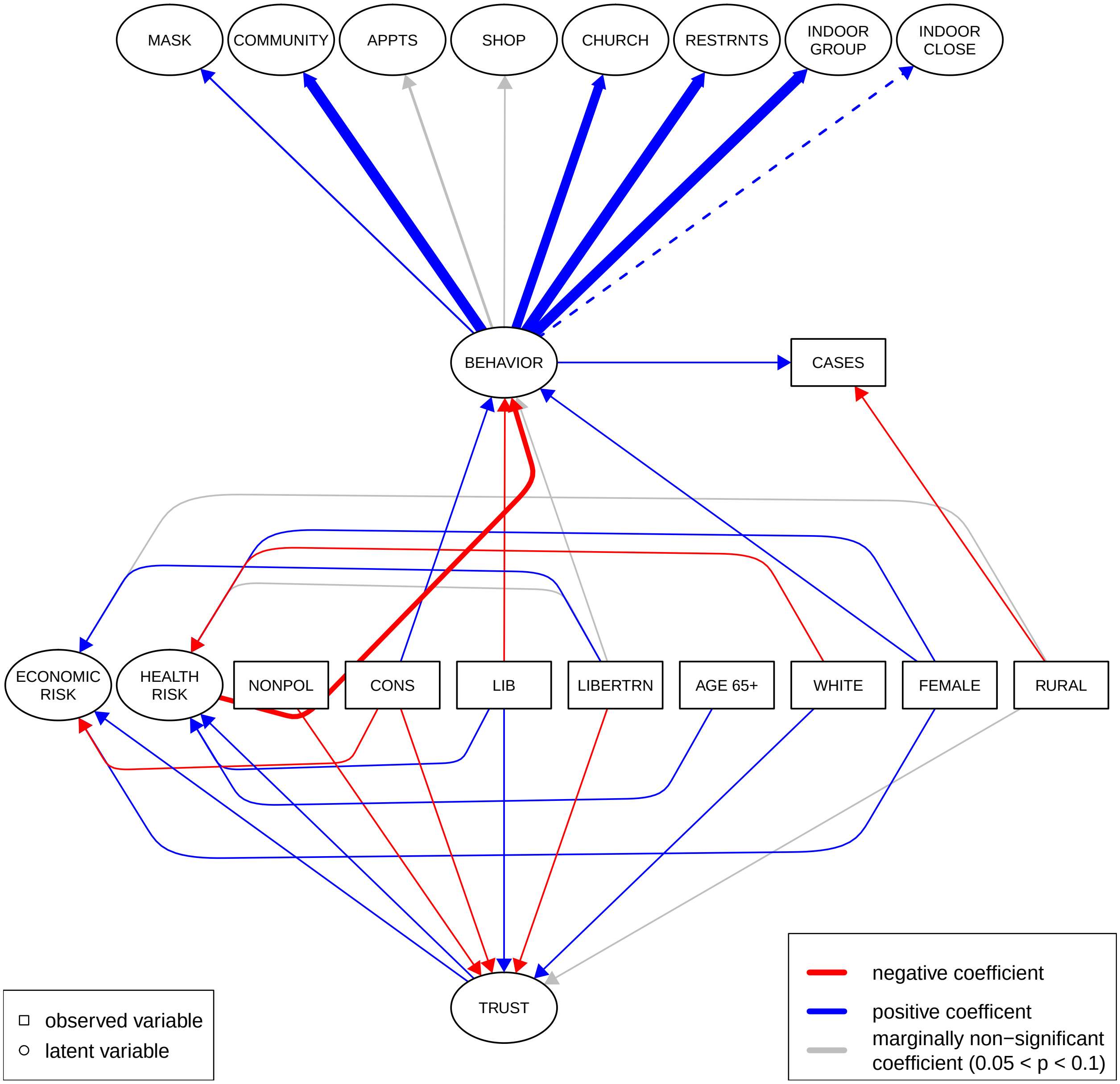

Figure S3.5: Model 1A - Trust to Risk Perception on Disease Burden Simplified Diagram

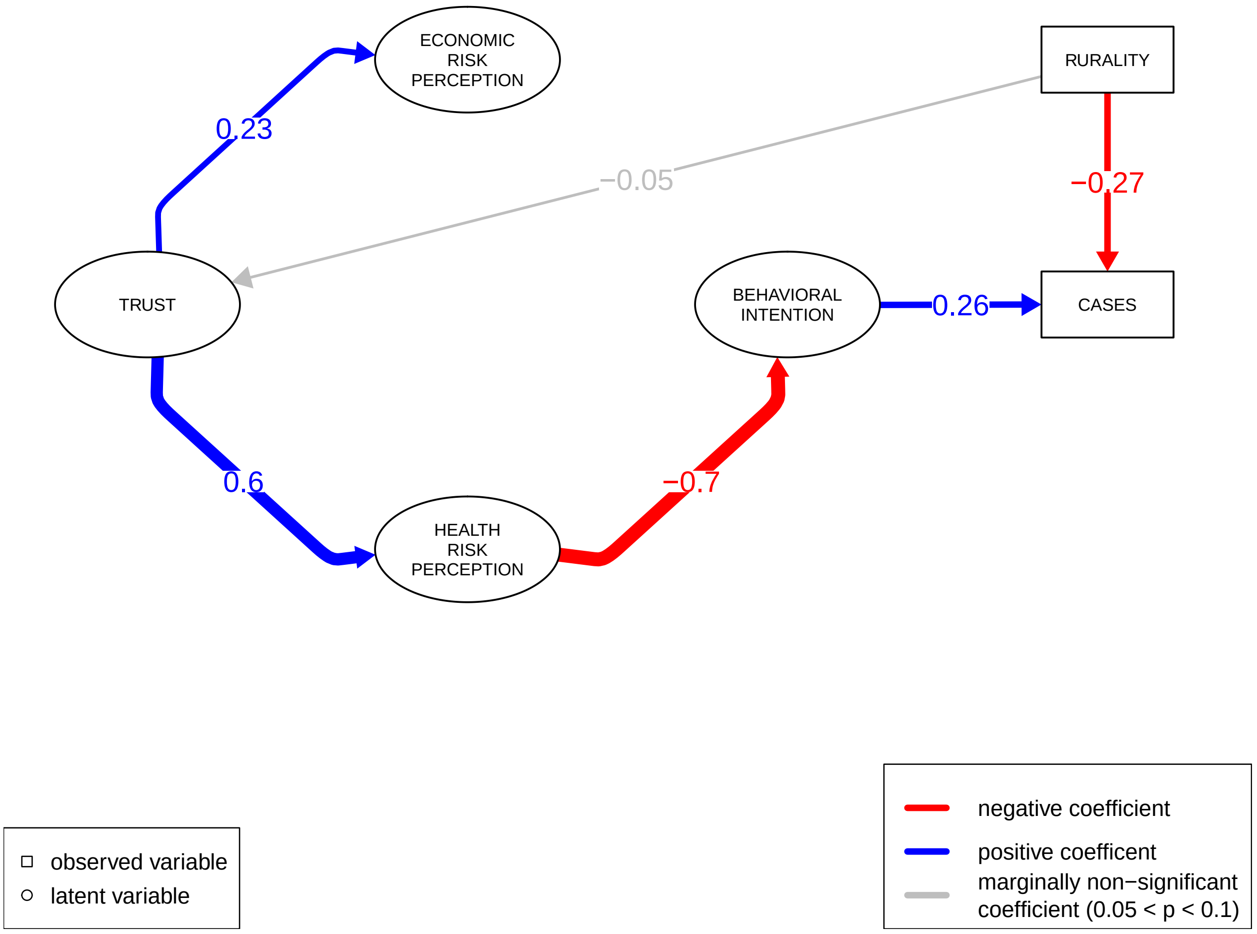

Table S3.5: Model 2A Risk Perception to Trust on Disease Burden Latent Table Output

Latent Variable	Regression Variable	std.est	std.error	z	p-value
trust	Q26_1	0.4341440	NA	NA	0.4341191
	Q26_2	0.6980109	11.801	0.000	0.6985734
	Q26_3	0.5273397	10.409	0.000	0.5274456
	Q26_5	0.6262664	11.294	0.000	0.6265990
	Q26_6	0.7717797	12.227	0.000	0.7726440
	Q26_7	0.8348637	12.527	0.000	0.8360459
	Q26_8	0.7860156	12.300	0.000	0.7869466
health_risk	Q2_1	0.8183126	NA	NA	0.8178201
	Q2_2	0.8078986	26.480	0.000	0.8074114
	Q2_3	0.7506042	23.963	0.000	0.7501516
	Q3_1	0.7839263	25.408	0.000	0.7834537
	Q3_5	0.7413485	23.571	0.000	0.7409016
	Q3_6	0.7604753	24.385	0.000	0.7600168
econ_risk	Q3_2	0.3702401	NA	NA	0.3700168
	Q3_3	0.5232499	8.308	0.000	0.5229341
	Q3_4	0.8330632	8.690	0.000	0.8325605
	Q3_7	0.5602534	8.518	0.000	0.5599156
indoor_close	Q4_1	0.0970931	NA	NA	0.0970350
	Q5_1	-0.7881713	-2.640	0.008	-0.7879046
	Q6_1	-0.8738406	-2.643	0.008	-0.8735972
	Q7_1	-0.8318297	-2.642	0.008	-0.8315719
	Q8_1	-0.5103893	-2.613	0.009	-0.5101389
indoor_group	Q4_2	0.2115848	NA	NA	0.2114650
	Q5_2	-0.6252361	-5.784	0.000	-0.6250645
	Q6_2	-0.8808590	-5.950	0.000	-0.8809030
	Q7_2	-0.9105587	-5.960	0.000	-0.9106451
	Q8_2	-0.7522777	-5.886	0.000	-0.7521806
restaurant	Q4_3	0.1357286	NA	NA	0.1356488
	Q5_3	-0.7013099	-3.727	0.000	-0.7011742
	Q6_3	-0.8125700	-3.745	0.000	-0.8125248
	Q7_3	-0.8931450	-3.753	0.000	-0.8931980
	Q8_3	-0.7600257	-3.737	0.000	-0.7599293
church	Q4_4	0.5070048	NA	NA	0.5067978
	Q5_4	-0.7297817	-14.282	0.000	-0.7296358
	Q6_4	-0.8800324	-15.518	0.000	-0.8800178
	Q7_4	-0.9207984	-15.766	0.000	-0.9208345
	Q8_4	-0.8002073	-14.914	0.000	-0.8001127
shop	Q4_5	0.0852857	NA	NA	0.0852344
	Q5_5	-0.5480449	-2.247	0.025	-0.5477548
	Q6_5	-0.7212224	-2.258	0.024	-0.7208804
	Q7_5	-0.8681273	-2.261	0.024	-0.8677630
	Q8_5	-0.6323104	-2.254	0.024	-0.6319910
appointments	Q4_6	0.0914998	NA	NA	0.0914449
	Q5_6	-0.5898646	-2.428	0.015	-0.5895998
	Q6_6	-0.7462555	-2.439	0.015	-0.7459902
	Q7_6	-0.8617853	-2.442	0.015	-0.8615480
	Q8_6	-0.6470381	-2.433	0.015	-0.6467681
community	Q4_7	0.1401801	NA	NA	0.1400978
	Q5_7	-0.6471119	-3.880	0.000	-0.6469466

(continued)

Latent Variable	Regression Variable	std.est	std.error	z	p-value
mask	Q6_7	-0.8673818	-3.921	0.000	-0.8673989
	Q7_7	-0.9293551	-3.927	0.000	-0.9294604
	Q8_7	-0.7816763	-3.909	0.000	-0.7816017
	Q5_8	0.8301274	NA	NA	0.8298714
	Q6_8	0.9368990	35.552	0.000	0.9366859
	Q7_8	0.9305476	35.205	0.000	0.9303312
	Q8_8	0.7495022	25.089	0.000	0.7492303

Table S3.6: Model 2A Risk Perception to Trust on Disease Burden Summary Table Output

Dependent Variables	Regression Variable	std.est	std.error	z	p-value
trust	ruralbool	-0.0461582	0.023	-1.733	0.083
	femalebool	-0.0271581	0.024	-1.003	0.316
	whitebool	0.1472786	0.041	5.111	0.000
	age65plusbool	-0.0898629	0.026	-3.167	0.002
	polVecLiberal	0.0398757	0.030	1.276	0.202
	polVecConservative	-0.2976379	0.039	-7.371	0.000
	polVecLibertarian	-0.1452722	0.069	-4.790	0.000
	polVecNonpolitical	-0.0991984	0.049	-3.371	0.001
	polVecOther	-0.0421418	0.085	-1.566	0.117
	health_risk	0.5658121	0.029	10.215	0.000
	econ_risk	-0.0276393	0.037	-0.874	0.382
health_risk	ruralbool	-0.0145403	0.052	-0.461	0.645
	femalebool	0.1362374	0.052	4.264	0.000
	whitebool	-0.0498518	0.087	-1.562	0.118
	age65plusbool	0.2228255	0.056	6.896	0.000
	polVecLiberal	0.1577016	0.067	4.276	0.000
	polVecConservative	-0.3373801	0.069	-8.933	0.000
	polVecLibertarian	-0.2231336	0.143	-6.724	0.000
	polVecNonpolitical	-0.0483513	0.107	-1.416	0.157
	polVecOther	0.0255556	0.191	0.797	0.426
econ_risk	ruralbool	-0.0787981	0.029	-2.003	0.045
	femalebool	0.0915440	0.030	2.295	0.022
	whitebool	-0.0353964	0.048	-0.906	0.365
	age65plusbool	0.0088206	0.031	0.227	0.821
	polVecLiberal	0.0775195	0.037	1.706	0.088
	polVecConservative	-0.2533050	0.043	-4.861	0.000
	polVecLibertarian	0.0438878	0.078	1.091	0.275
	polVecNonpolitical	-0.0378155	0.059	-0.905	0.365
	polVecOther	0.0133654	0.105	0.342	0.733
beh_int	trust	-0.6246913	0.034	-2.550	0.011
	ruralbool	-0.0202233	0.003	-0.752	0.452
	femalebool	0.0147160	0.003	0.555	0.579
	whitebool	0.0706311	0.007	1.878	0.060
	age65plusbool	-0.1449152	0.008	-2.376	0.017
	polVecLiberal	-0.1211492	0.007	-2.198	0.028
	polVecConservative	0.1573775	0.009	2.274	0.023
	polVecLibertarian	0.0496342	0.011	1.456	0.145
	polVecNonpolitical	-0.0209865	0.007	-0.718	0.473
	polVecOther	-0.0251222	0.012	-0.904	0.366
cumcasesper100	beh_int	0.2872415	1.915	2.511	0.012
	ruralbool	-0.2734161	0.065	-8.518	0.000

Note:

chisq = 10648.493 aic = 98950.456 rmsea = 0.072

Figure S3.6: Model 2A - Risk Perception to Trust on Disease Burden Full Diagram

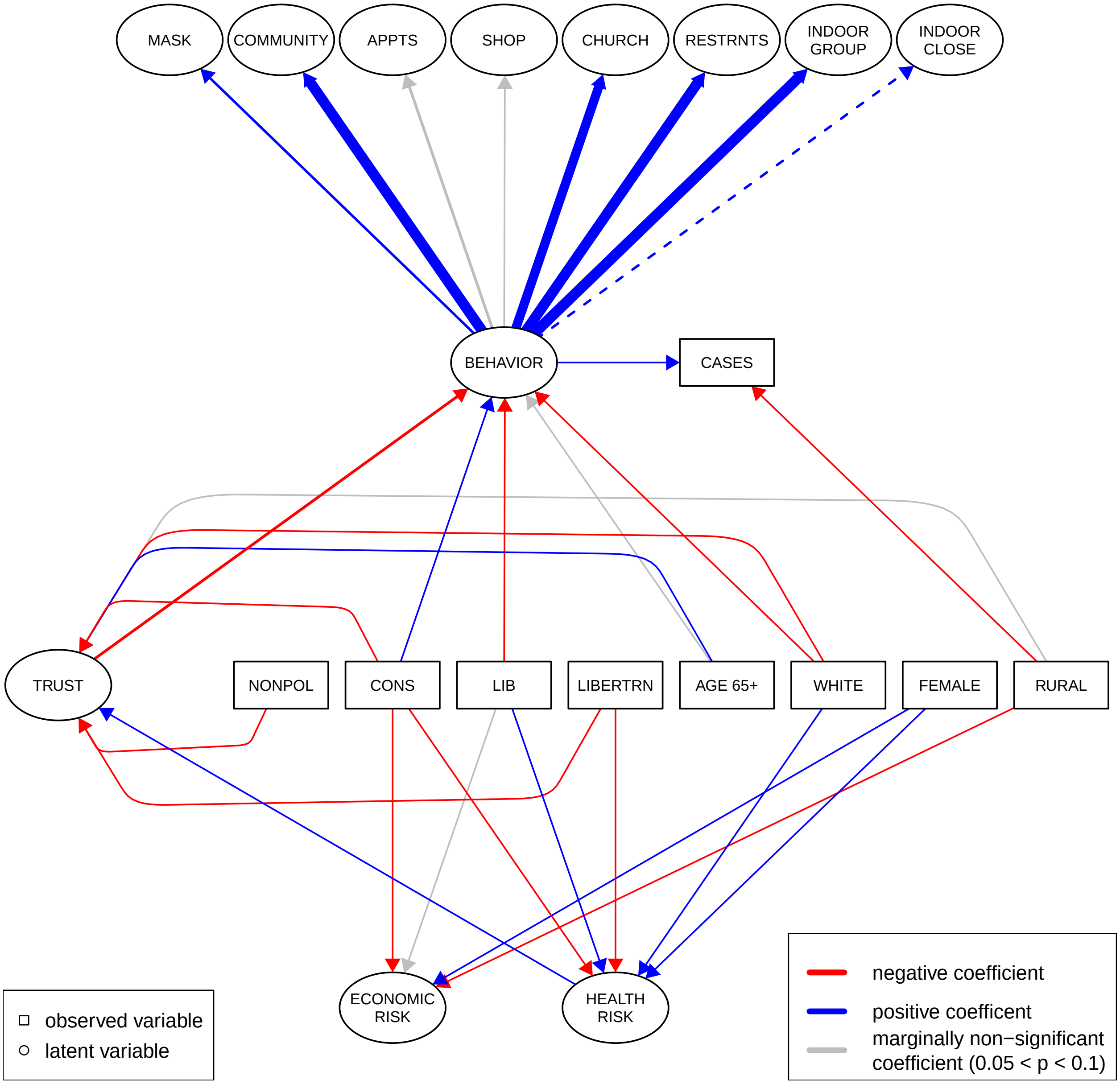

Figure S3.7: Model 2A - Risk Perception to Trust on Disease Burden Simplified Diagram

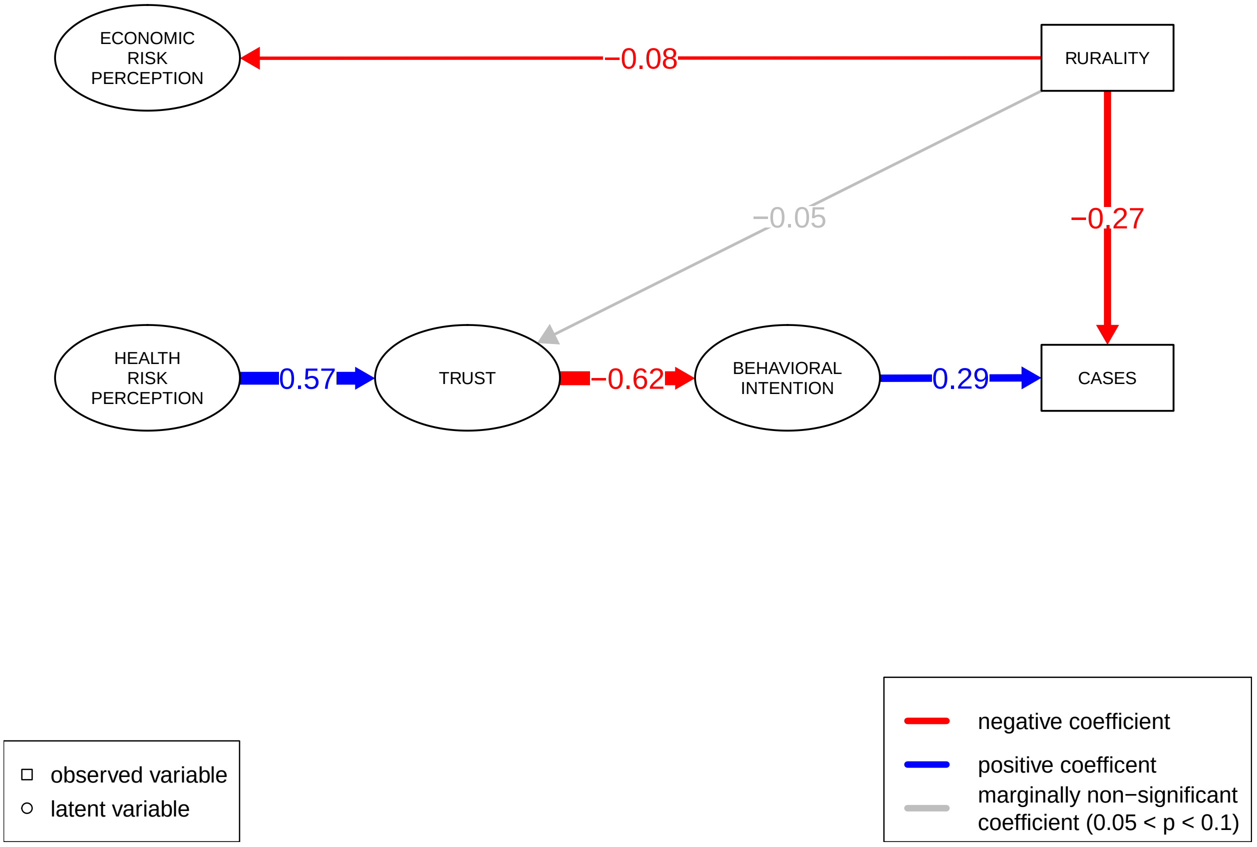

Table S3.7: Model 3A Trust and Risk Perception on Disease Burden Latent Table Output

Latent Variable	Regression Variable	std.est	std.error	z	p-value
trust	Q26_1	0.4745503	NA	NA	0.4742630
	Q26_2	0.7160696	12.899	0.000	0.7156374
	Q26_3	0.5557654	11.365	0.000	0.5554304
	Q26_5	0.6433199	12.276	0.000	0.6429328
	Q26_6	0.7668329	13.270	0.000	0.7663695
	Q26_7	0.8153349	13.579	0.000	0.8148424
	Q26_8	0.7913032	13.432	0.000	0.7908258
health_risk	Q2_1	0.8350712	NA	NA	0.8345678
	Q2_2	0.8186568	27.856	0.000	0.8181628
	Q2_3	0.7467118	24.405	0.000	0.7462613
	Q3_1	0.7930764	26.590	0.000	0.7925980
	Q3_5	0.7210158	23.255	0.000	0.7205807
	Q3_6	0.7386590	24.040	0.000	0.7382132
econ_risk	Q3_2	0.3682274	NA	NA	0.3680054
	Q3_3	0.5235169	8.283	0.000	0.5232012
	Q3_4	0.8342364	8.665	0.000	0.8337333
	Q3_7	0.5598192	8.487	0.000	0.5594807
indoor_close	Q4_1	0.0953896	NA	NA	0.0953203
	Q5_1	-0.7837273	-2.589	0.010	-0.7767508
	Q6_1	-0.8702911	-2.592	0.010	-0.8608821
	Q7_1	-0.8282499	-2.591	0.010	-0.8200826
	Q8_1	-0.5071693	-2.563	0.010	-0.5050879
indoor_group	Q4_2	0.2056817	NA	NA	0.2053276
	Q5_2	-0.6142134	-5.603	0.000	-0.6077969
	Q6_2	-0.8772771	-5.764	0.000	-0.8593965
	Q7_2	-0.9049676	-5.772	0.000	-0.8854108
	Q8_2	-0.7430620	-5.702	0.000	-0.7319824
restaurant	Q4_3	0.1319234	NA	NA	0.1317835
	Q5_3	-0.6920554	-3.612	0.000	-0.6831025
	Q6_3	-0.8053507	-3.629	0.000	-0.7915010
	Q7_3	-0.8877862	-3.637	0.000	-0.8694434
	Q8_3	-0.7517928	-3.622	0.000	-0.7404336
church	Q4_4	0.4978154	NA	NA	0.4946099
	Q5_4	-0.7221650	-13.917	0.000	-0.7129425
	Q6_4	-0.8761296	-15.119	0.000	-0.8600441
	Q7_4	-0.9165468	-15.348	0.000	-0.8982276
	Q8_4	-0.7929431	-14.524	0.000	-0.7808762
shop	Q4_5	0.0842204	NA	NA	0.0841650
	Q5_5	-0.5456078	-2.215	0.027	-0.5440276
	Q6_5	-0.7196876	-2.226	0.026	-0.7163905
	Q7_5	-0.8647526	-2.229	0.026	-0.8592768
	Q8_5	-0.6298825	-2.221	0.026	-0.6275802
appointments	Q4_6	0.0891118	NA	NA	0.0890484
	Q5_6	-0.5855164	-2.359	0.018	-0.5824275
	Q6_6	-0.7422689	-2.369	0.018	-0.7362716
	Q7_6	-0.8573960	-2.372	0.018	-0.8483583
	Q8_6	-0.6421072	-2.363	0.018	-0.6381166
community	Q4_7	0.1360673	NA	NA	0.1359189
	Q5_7	-0.6380892	-3.757	0.000	-0.6309797

(continued)

Latent Variable	Regression Variable	std.est	std.error	z	p-value
mask	Q6_7	-0.8625894	-3.796	0.000	-0.8456725
	Q7_7	-0.9248190	-3.801	0.000	-0.9041384
	Q8_7	-0.7732857	-3.784	0.000	-0.7609387
	Q5_8	0.8265691	NA	NA	0.8186493
	Q6_8	0.9353734	35.022	0.000	0.9240949
	Q7_8	0.9289274	34.687	0.000	0.9178703
	Q8_8	0.7446758	24.711	0.000	0.7387858

Table S3.8: Model 3A Trust and Risk Perception on Disease Burden Summary Table Output

Dependent Variables	Regression Variable	std.est	std.error	z	p-value
trust	ruralbool	-0.0504009	0.030	-1.633	0.103
	femalebool	0.0450828	0.030	1.452	0.146
	whitebool	0.1237901	0.050	3.856	0.000
	age65plusbool	0.0380944	0.032	1.225	0.221
	polVecLiberal	0.1261649	0.039	3.441	0.001
	polVecConservative	-0.4703305	0.050	-9.834	0.000
	polVecLibertarian	-0.2694991	0.092	-7.355	0.000
	polVecNonpolitical	-0.1249602	0.062	-3.653	0.000
	polVecOther	-0.0295334	0.108	-0.945	0.344
health_risk	ruralbool	-0.0129802	0.053	-0.410	0.682
	femalebool	0.1338093	0.054	4.184	0.000
	whitebool	-0.0507499	0.088	-1.587	0.113
	age65plusbool	0.2272469	0.058	7.029	0.000
	polVecLiberal	0.1562608	0.068	4.232	0.000
	polVecConservative	-0.3317360	0.070	-8.800	0.000
	polVecLibertarian	-0.2203727	0.146	-6.641	0.000
	polVecNonpolitical	-0.0452897	0.109	-1.324	0.185
	polVecOther	0.0289099	0.195	0.899	0.369
econ_risk	ruralbool	-0.0789176	0.029	-2.007	0.045
	femalebool	0.0914534	0.029	2.294	0.022
	whitebool	-0.0353031	0.048	-0.904	0.366
	age65plusbool	0.0088372	0.031	0.227	0.820
	polVecLiberal	0.0774280	0.037	1.705	0.088
	polVecConservative	-0.2530092	0.043	-4.853	0.000
	polVecLibertarian	0.0440567	0.078	1.096	0.273
	polVecNonpolitical	-0.0379060	0.059	-0.908	0.364
	polVecOther	0.0132560	0.105	0.339	0.735
beh_int	econ_risk	0.0570876	0.006	1.567	0.117
	health_risk	-0.5859893	0.016	-2.550	0.011
	trust	-0.3008314	0.015	-2.450	0.014
	ruralbool	-0.0056390	0.003	-0.237	0.813
	femalebool	0.0709680	0.004	1.935	0.053
	whitebool	0.0051738	0.005	0.213	0.831
	age65plusbool	-0.0295435	0.003	-1.082	0.279
	polVecLiberal	-0.0830591	0.006	-1.941	0.052
	polVecConservative	0.1538314	0.009	2.243	0.025
	polVecLibertarian	0.0154552	0.008	0.563	0.573
	polVecNonpolitical	-0.0024890	0.006	-0.096	0.923
	polVecOther	-0.0005295	0.010	-0.022	0.982
cumcasesper100	beh_int	0.2600091	1.816	2.443	0.015
cumcasesper100	ruralbool	-0.2737575	0.065	-8.461	0.000

Note:

chisq = 10629.99 aic = 98931.952 rmsea = 0.072

Figure S3.8: Model 3A - Trust and Risk Perception on Disease Burden Full Diagram

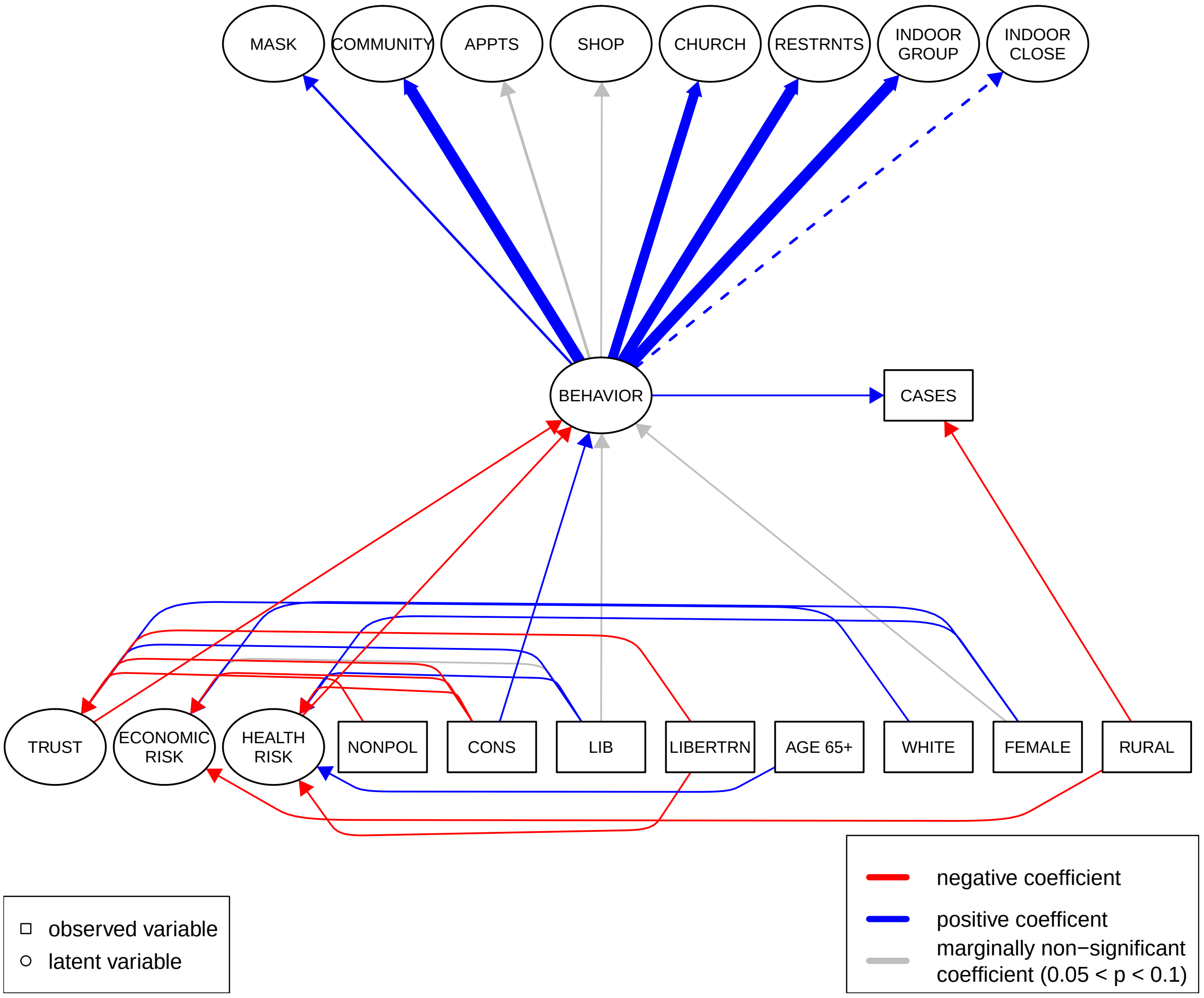

Figure S3.9: Model 3A - Trust and Risk Perception on Disease Burden Simplified Diagram

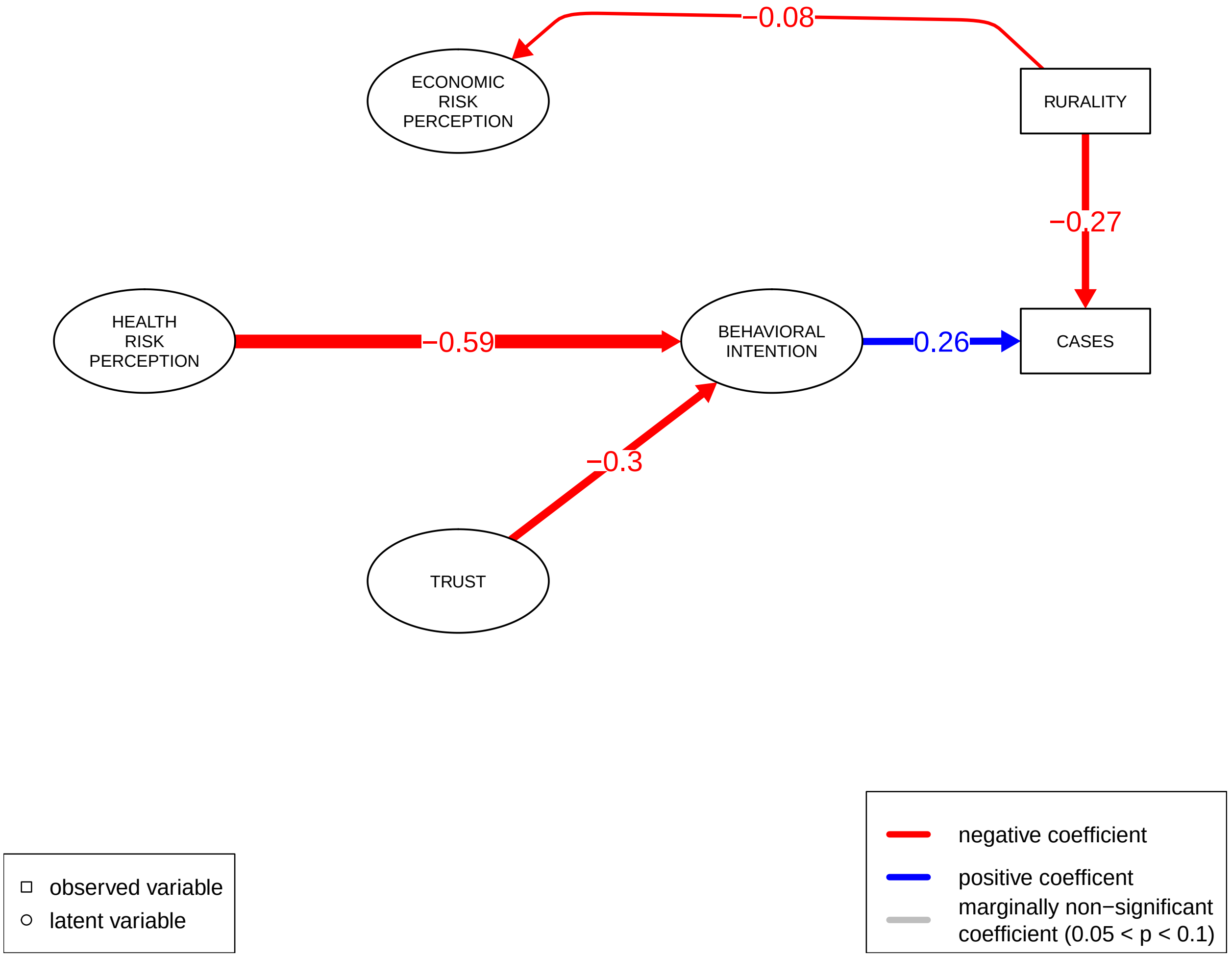

Table S3.9: Model 1B - Trust to Risk Perception on Behavioral Intention Latent Table Output

Latent Variable	Regression Variable	std.est	std.error	z	p-value
trust	Q26_1	0.4395254	0.000	NA	NA
	Q26_2	0.6953264	0.133	11.873	0.000
	Q26_3	0.5369483	0.116	10.561	0.000
	Q26_5	0.6374691	0.127	11.457	0.000
	Q26_6	0.7721043	0.142	12.331	0.000
	Q26_7	0.8354558	0.150	12.639	0.000
	Q26_8	0.7941464	0.145	12.445	0.000
health_risk	Q2_1	0.8210521	0.000	NA	NA
	Q2_2	0.8129030	0.036	27.130	0.000
	Q2_3	0.7461942	0.038	24.081	0.000
	Q3_1	0.7850996	0.037	25.827	0.000
	Q3_5	0.7300594	0.038	23.383	0.000
	Q3_6	0.7555805	0.038	24.494	0.000
econ_risk	Q3_2	0.3820106	0.000	NA	NA
	Q3_3	0.5454562	0.168	8.522	0.000
	Q3_4	0.8042947	0.235	8.967	0.000
	Q3_7	0.5658953	0.172	8.636	0.000
indoor_close	Q4_1	0.0967933	0.000	NA	NA
	Q5_1	-0.7890589	3.103	-2.633	0.008
	Q6_1	-0.8739740	3.435	-2.636	0.008
	Q7_1	-0.8326451	3.273	-2.635	0.008
	Q8_1	-0.5138147	2.038	-2.607	0.009
indoor_group	Q4_2	0.2122915	0.000	NA	NA
	Q5_2	-0.6267978	0.510	-5.807	0.000
	Q6_2	-0.8838765	0.701	-5.974	0.000
	Q7_2	-0.9105001	0.721	-5.983	0.000
	Q8_2	-0.7535910	0.603	-5.910	0.000
restaurant	Q4_3	0.1358494	0.000	NA	NA
	Q5_3	-0.7033298	1.391	-3.734	0.000
	Q6_3	-0.8144830	1.606	-3.752	0.000
	Q7_3	-0.8940728	1.760	-3.760	0.000
	Q8_3	-0.7625969	1.505	-3.745	0.000
church	Q4_4	0.5088616	0.000	NA	NA
	Q5_4	-0.7323200	0.100	-14.367	0.000
	Q6_4	-0.8823713	0.111	-15.611	0.000
	Q7_4	-0.9210007	0.115	-15.850	0.000
	Q8_4	-0.8011326	0.105	-14.991	0.000
shop	Q4_5	0.0848159	0.000	NA	NA
	Q5_5	-0.5493870	2.900	-2.235	0.025
	Q6_5	-0.7232467	3.801	-2.246	0.025
	Q7_5	-0.8668014	4.552	-2.249	0.025
	Q8_5	-0.6337765	3.337	-2.241	0.025
appointments	Q4_6	0.0904093	0.000	NA	NA
	Q5_6	-0.5923514	2.733	-2.401	0.016
	Q6_6	-0.7481126	3.440	-2.411	0.016
	Q7_6	-0.8611330	3.957	-2.414	0.016
	Q8_6	-0.6489136	2.989	-2.405	0.016
community	Q4_7	0.1405399	0.000	NA	NA
	Q5_7	-0.6507521	1.193	-3.893	0.000

(continued)

Latent Variable	Regression Variable	std.est	std.error	z	p-value
	Q6_7	-0.8696896	1.582	-3.933	0.000
	Q7_7	-0.9295026	1.690	-3.939	0.000
	Q8_7	-0.7827925	1.427	-3.921	0.000
mask	Q5_8	0.8309243	0.000	NA	NA
	Q6_8	0.9374471	0.032	35.689	0.000
	Q7_8	0.9309682	0.032	35.331	0.000
	Q8_8	0.7502065	0.036	25.159	0.000

Table S3.10: Model 1B - Trust to Risk Perception on Behavioral Intention Summary Table Output

Dependent Variables	Regression Variable	std.est	std.error	z	p-value
trust	ruralbool	-0.0524177	0.027	-1.709	0.088
	femalebool	0.0471707	0.027	1.529	0.126
	whitebool	0.1198171	0.047	3.744	0.000
	age65plusbool	0.0355333	0.029	1.152	0.250
	polVecLiberal	0.1269346	0.036	3.468	0.001
	polVecConservative	-0.4792851	0.049	-9.542	0.000
	polVecLibertarian	-0.2718985	0.087	-7.262	0.000
	polVecNonpolitical	-0.1254447	0.057	-3.671	0.000
	polVecOther	-0.0278327	0.099	-0.898	0.369
health_risk	trust	0.5973360	0.112	9.939	0.000
	ruralbool	0.0174238	0.047	0.622	0.534
	femalebool	0.1073022	0.047	3.791	0.000
	whitebool	-0.1218331	0.078	-4.238	0.000
	age65plusbool	0.2030798	0.050	7.091	0.000
	polVecLiberal	0.0818772	0.060	2.488	0.013
	polVecConservative	-0.0504230	0.069	-1.341	0.180
	polVecLibertarian	-0.0606063	0.134	-1.962	0.050
	polVecNonpolitical	0.0273616	0.096	0.894	0.371
	polVecOther	0.0432130	0.170	1.520	0.129
econ_risk	trust	0.2290899	0.052	3.819	0.000
	ruralbool	-0.0656218	0.030	-1.672	0.094
	femalebool	0.0837533	0.030	2.103	0.035
	whitebool	-0.0643906	0.051	-1.609	0.108
	age65plusbool	0.0035750	0.032	0.092	0.927
	polVecLiberal	0.0507565	0.039	1.112	0.266
	polVecConservative	-0.1511760	0.046	-2.804	0.005
	polVecLibertarian	0.0995460	0.088	2.278	0.023
	polVecNonpolitical	-0.0080773	0.062	-0.191	0.848
	polVecOther	0.0210756	0.109	0.536	0.592
beh_int	econ_risk	0.0709011	0.006	1.790	0.073
	health_risk	-0.7169805	0.021	-2.602	0.009
	ruralbool	0.0380051	0.003	1.372	0.170
	femalebool	0.0649470	0.004	1.905	0.057
	whitebool	-0.0308915	0.005	-1.191	0.234
	age65plusbool	0.0007897	0.003	0.033	0.974
	polVecLiberal	-0.0563036	0.005	-1.613	0.107
	polVecConservative	0.2140480	0.012	2.469	0.014
	polVecLibertarian	0.0564483	0.011	1.726	0.084
	polVecNonpolitical	0.0281905	0.006	1.047	0.295
	polVecOther	0.0121386	0.010	0.514	0.607
	casetodateper100	0.1280073	0.002	2.351	0.019

Note:

chisq = 10336.253 aic = 89908.147 rmsea = 0.071

Figure S3.10: Model 1B - Trust to Risk Perception on Behavioral Intention Full Diagram

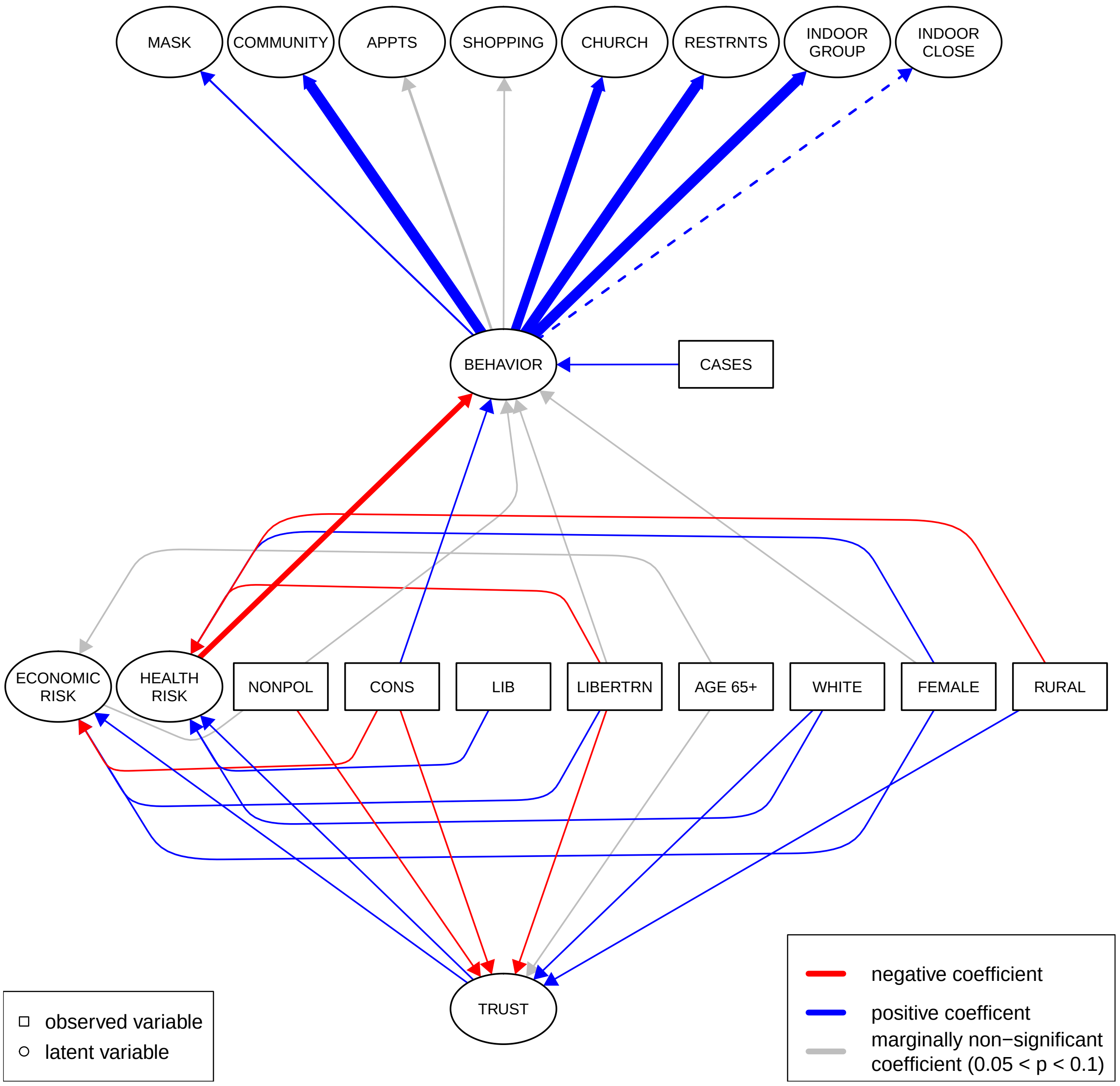

Figure S3.11: Model 1B - Trust to Risk Perception on Behavioral Intention Simplified Diagram

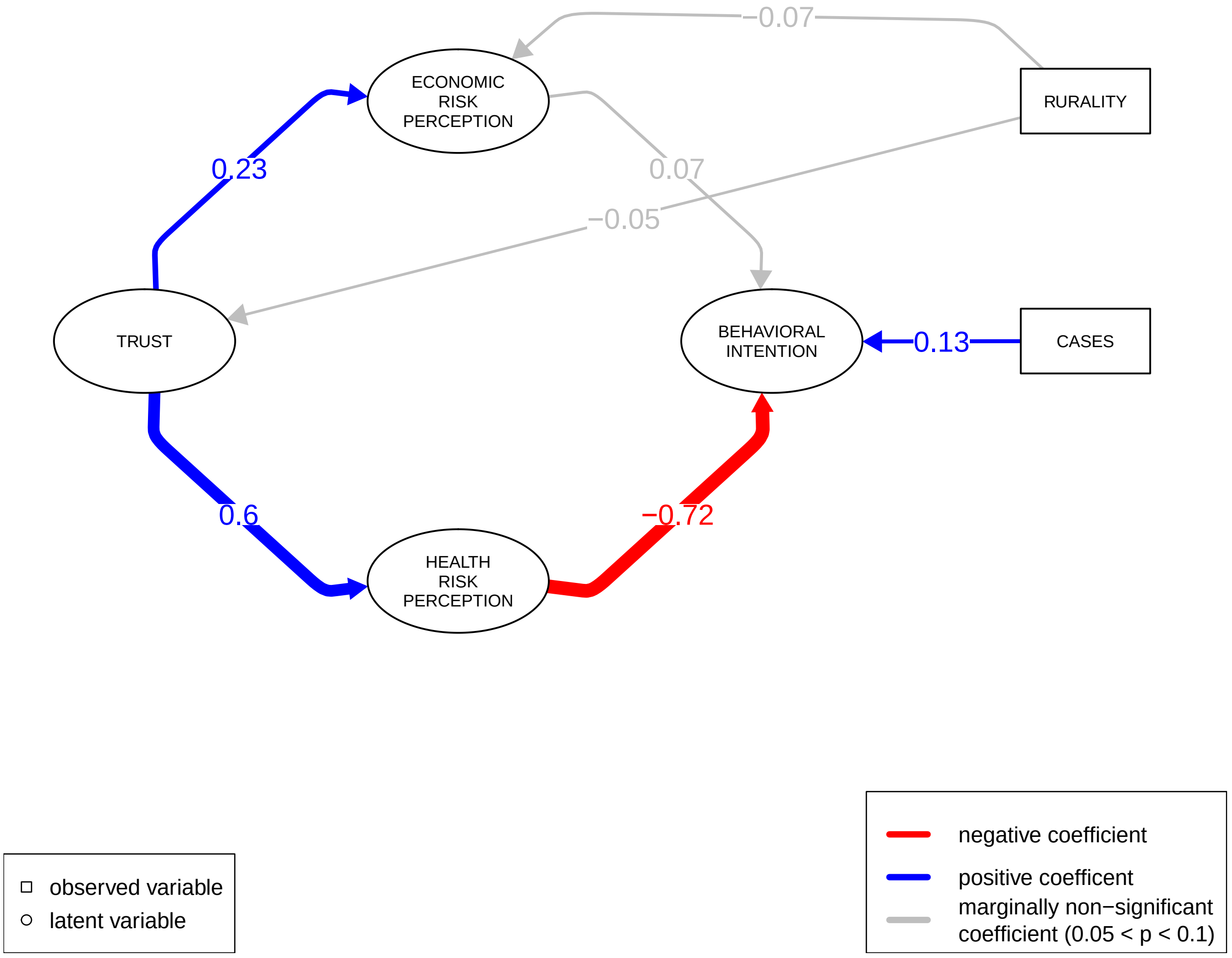

Table S3.11: Model 2B - Risk Perception to Trust on Behavioral Intention Latent Table Ouput

Latent Variable	Regression Variable	std.est	std.error	z	p-value
trust	Q26_1	0.4305537	0.000	NA	NA
	Q26_2	0.6957501	0.138	11.691	0.000
	Q26_3	0.5254561	0.118	10.324	0.000
	Q26_5	0.6264508	0.130	11.211	0.000
	Q26_6	0.7723280	0.148	12.124	0.000
	Q26_7	0.8362713	0.157	12.418	0.000
	Q26_8	0.7862412	0.150	12.193	0.000
health_risk	Q2_1	0.8182902	0.000	NA	NA
	Q2_2	0.8078837	0.037	26.480	0.000
	Q2_3	0.7506048	0.038	23.963	0.000
	Q3_1	0.7838617	0.038	25.406	0.000
	Q3_5	0.7414424	0.038	23.575	0.000
	Q3_6	0.7604973	0.038	24.387	0.000
econ_risk	Q3_2	0.3702177	0.000	NA	NA
	Q3_3	0.5231506	0.170	8.308	0.000
	Q3_4	0.8331993	0.259	8.690	0.000
	Q3_7	0.5601998	0.178	8.518	0.000
indoor_close	Q4_1	0.0972684	0.000	NA	NA
	Q5_1	-0.7881721	3.064	-2.644	0.008
	Q6_1	-0.8739118	3.393	-2.648	0.008
	Q7_1	-0.8313852	3.230	-2.646	0.008
	Q8_1	-0.5099787	2.003	-2.617	0.009
indoor_group	Q4_2	0.2110266	0.000	NA	NA
	Q5_2	-0.6244214	0.513	-5.768	0.000
	Q6_2	-0.8806234	0.703	-5.933	0.000
	Q7_2	-0.9102999	0.726	-5.943	0.000
	Q8_2	-0.7521907	0.607	-5.871	0.000
restaurant	Q4_3	0.1355116	0.000	NA	NA
	Q5_3	-0.7000439	1.388	-3.720	0.000
	Q6_3	-0.8112709	1.601	-3.738	0.000
	Q7_3	-0.8934126	1.759	-3.746	0.000
	Q8_3	-0.7604253	1.504	-3.731	0.000
church	Q4_4	0.5057873	0.000	NA	NA
	Q5_4	-0.7290669	0.101	-14.239	0.000
	Q6_4	-0.8795644	0.112	-15.468	0.000
	Q7_4	-0.9209789	0.116	-15.718	0.000
	Q8_4	-0.8001114	0.106	-14.872	0.000
shop	Q4_5	0.0853570	0.000	NA	NA
	Q5_5	-0.5481947	2.857	-2.248	0.025
	Q6_5	-0.7214139	3.740	-2.259	0.024
	Q7_5	-0.8674638	4.492	-2.262	0.024
	Q8_5	-0.6324333	3.286	-2.255	0.024
appointments	Q4_6	0.0912015	0.000	NA	NA
	Q5_6	-0.5895735	2.671	-2.420	0.016
	Q6_6	-0.7458301	3.364	-2.430	0.015
	Q7_6	-0.8613503	3.880	-2.434	0.015
	Q8_6	-0.6474415	2.928	-2.425	0.015
community	Q4_7	0.1398227	0.000	NA	NA
	Q5_7	-0.6463532	1.194	-3.870	0.000

(continued)

Latent Variable	Regression Variable	std.est	std.error	z	p-value
	Q6_7	-0.8664904	1.584	-3.911	0.000
	Q7_7	-0.9294705	1.697	-3.917	0.000
	Q8_7	-0.7819252	1.434	-3.899	0.000
mask	Q5_8	0.8300059	0.000	NA	NA
	Q6_8	0.9368420	0.032	35.532	0.000
	Q7_8	0.9304379	0.032	35.183	0.000
	Q8_8	0.7492649	0.036	25.072	0.000

Table S3.12: Model 2B - Risk Perception to Trust on Behavioral Intention Summary Table Ouput

Dependent Variables	Regression Variable	std.est	std.error	z	p-value
trust	ruralbool	-0.0463969	0.023	-1.745	0.081
	femalebool	-0.0271250	0.023	-1.003	0.316
	whitebool	0.1469592	0.041	5.101	0.000
	age65plusbool	-0.0903822	0.026	-3.187	0.001
	polVecLiberal	0.0397291	0.030	1.274	0.203
	polVecConservative	-0.2980511	0.039	-7.363	0.000
	polVecLibertarian	-0.1450684	0.069	-4.785	0.000
	polVecNonpolitical	-0.0991899	0.048	-3.374	0.001
	polVecOther	-0.0420054	0.084	-1.564	0.118
	health_risk	0.5672902	0.029	10.167	0.000
	econ_risk	-0.0285050	0.037	-0.903	0.367
health_risk	ruralbool	-0.0145495	0.052	-0.461	0.645
	femalebool	0.1362395	0.052	4.264	0.000
	whitebool	-0.0498488	0.087	-1.561	0.118
	age65plusbool	0.2228096	0.056	6.895	0.000
	polVecLiberal	0.1577046	0.067	4.276	0.000
	polVecConservative	-0.3373909	0.069	-8.933	0.000
	polVecLibertarian	-0.2231377	0.143	-6.724	0.000
	polVecNonpolitical	-0.0483584	0.107	-1.417	0.157
	polVecOther	0.0255500	0.191	0.796	0.426
econ_risk	ruralbool	-0.0788062	0.029	-2.004	0.045
	femalebool	0.0915249	0.030	2.295	0.022
	whitebool	-0.0353921	0.048	-0.906	0.365
	age65plusbool	0.0088043	0.031	0.226	0.821
	polVecLiberal	0.0775047	0.037	1.706	0.088
	polVecConservative	-0.2532709	0.043	-4.860	0.000
	polVecLibertarian	0.0439062	0.078	1.092	0.275
	polVecNonpolitical	-0.0378203	0.059	-0.906	0.365
	polVecOther	0.0133595	0.105	0.342	0.733
beh_int	trust	-0.6204804	0.034	-2.552	0.011
	ruralbool	-0.0123779	0.003	-0.456	0.648
	femalebool	0.0097836	0.003	0.371	0.711
	whitebool	0.0728175	0.008	1.900	0.057
	age65plusbool	-0.1450171	0.008	-2.376	0.018
	polVecLiberal	-0.1073202	0.007	-2.093	0.036
	polVecConservative	0.1544940	0.009	2.259	0.024
	polVecLibertarian	0.0533238	0.011	1.522	0.128
	polVecNonpolitical	-0.0185510	0.007	-0.636	0.525
	polVecOther	-0.0236812	0.012	-0.852	0.394
	casetodateper100	0.0304744	0.001	1.025	0.305

Note:

chisq = 10552.368 aic = 90122.262 rmsea = 0.072

Figure S3.14: Model 2B - Risk Perception to Trust on Behavioral Intention Full Diagram

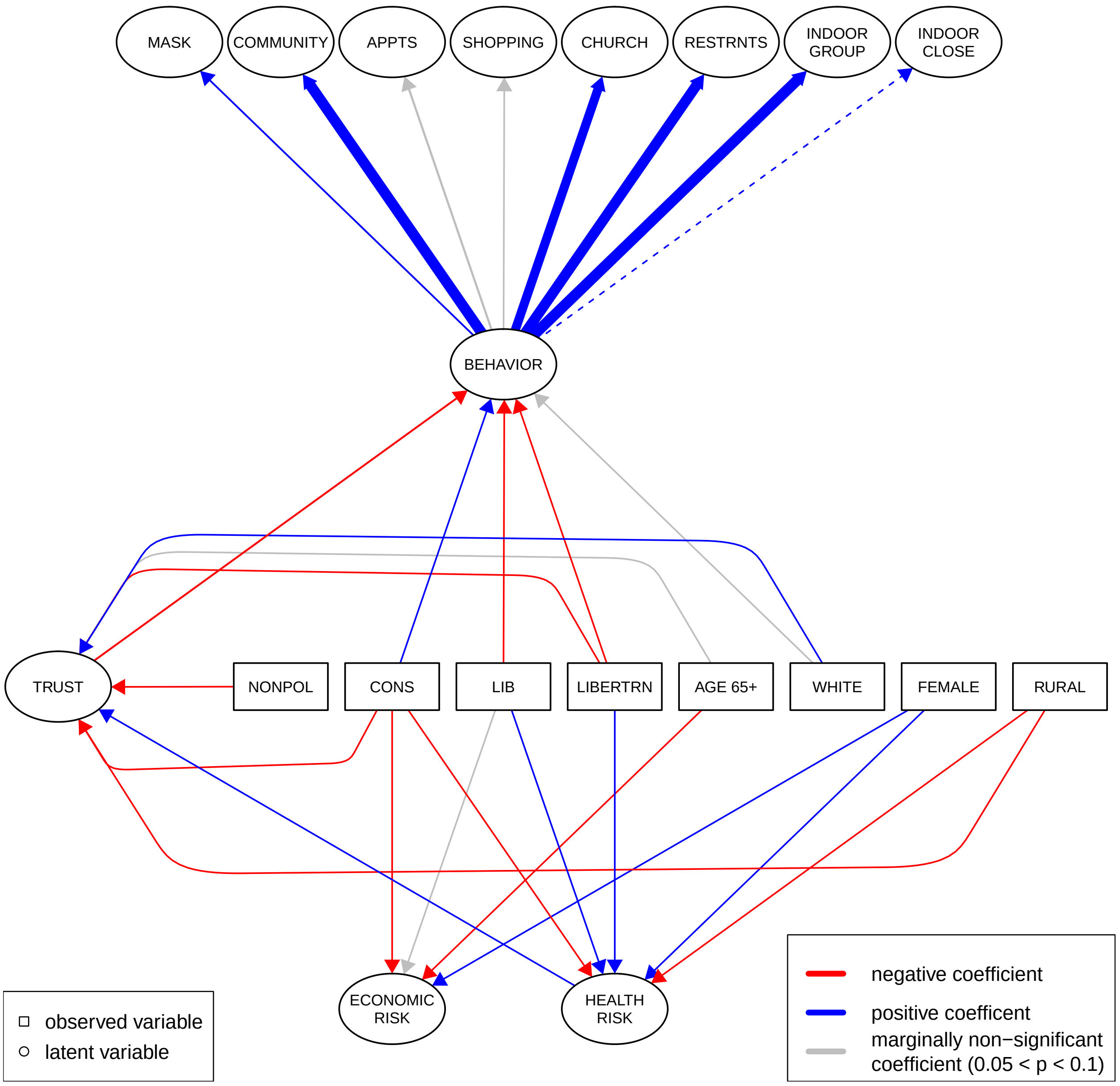

Figure S3.15: Model 2B - Risk Perception to Trust on Behavioral Intention Simplified Diagram

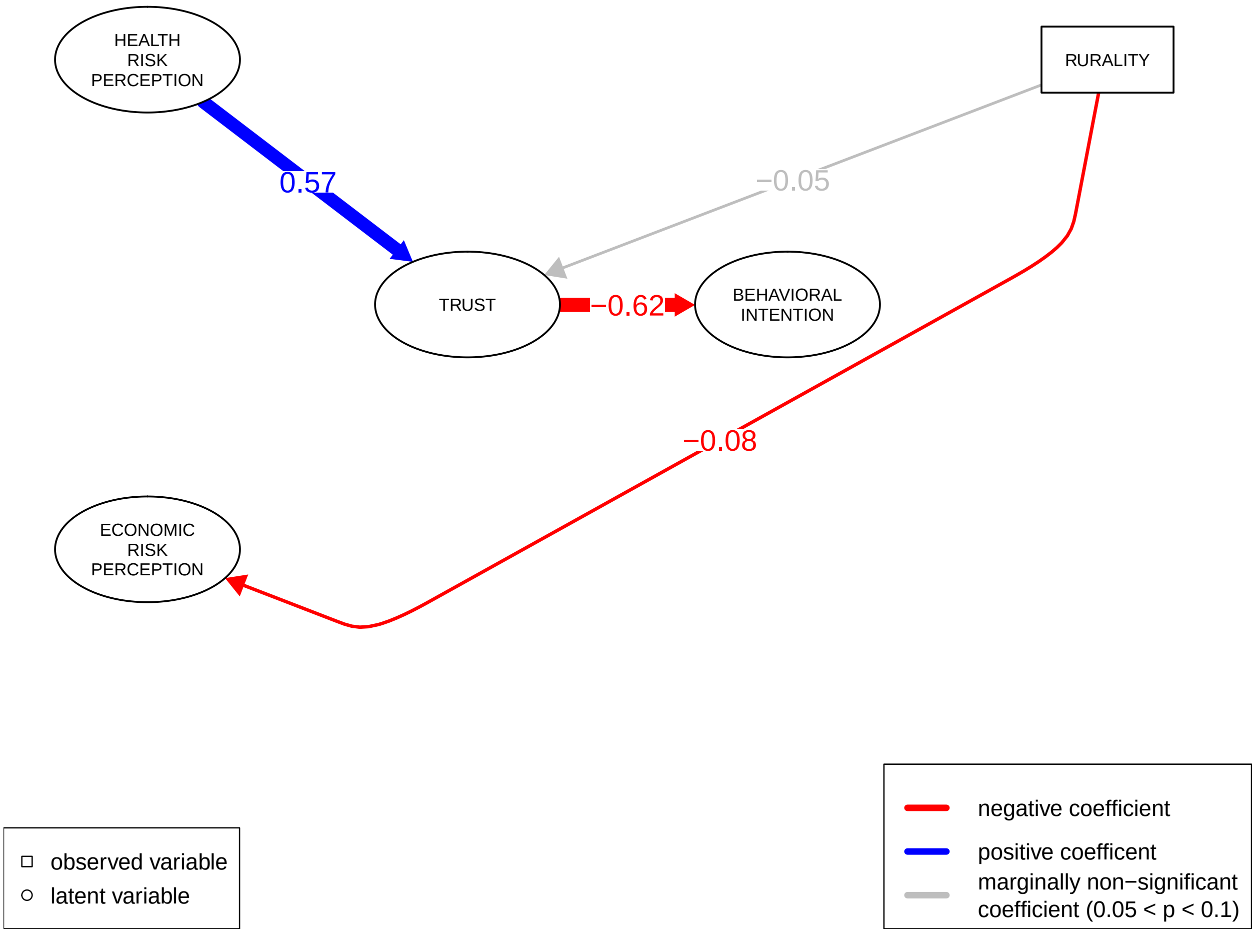

Table S3.13: Model 3B - Trust and Risk Perception on Behavioral Intention Latent Table Output

Latent Variable	Regression Variable	std.est	std.error	z	p-value
trust	Q26_1	0.4697184	0.000	NA	NA
	Q26_2	0.7125492	0.119	12.738	0.000
	Q26_3	0.5536324	0.105	11.249	0.000
	Q26_5	0.6442173	0.113	12.169	0.000
	Q26_6	0.7676961	0.124	13.131	0.000
	Q26_7	0.8176045	0.130	13.436	0.000
	Q26_8	0.7924331	0.127	13.288	0.000
health_risk	Q2_1	0.8358804	0.000	NA	NA
	Q2_2	0.8195140	0.035	27.979	0.000
	Q2_3	0.7464089	0.037	24.445	0.000
	Q3_1	0.7926002	0.036	26.635	0.000
	Q3_5	0.7209042	0.037	23.298	0.000
	Q3_6	0.7373750	0.037	24.034	0.000
econ_risk	Q3_2	0.3673182	0.000	NA	NA
	Q3_3	0.5222315	0.172	8.271	0.000
	Q3_4	0.8360688	0.263	8.656	0.000
	Q3_7	0.5594285	0.180	8.479	0.000
indoor_close	Q4_1	0.0955903	0.000	NA	NA
	Q5_1	-0.7845838	3.141	-2.595	0.009
	Q6_1	-0.8708465	3.477	-2.598	0.009
	Q7_1	-0.8286462	3.313	-2.597	0.009
	Q8_1	-0.5078572	2.062	-2.569	0.010
indoor_group	Q4_2	0.2064242	0.000	NA	NA
	Q5_2	-0.6154866	0.526	-5.626	0.000
	Q6_2	-0.8781783	0.723	-5.788	0.000
	Q7_2	-0.9056641	0.744	-5.796	0.000
	Q8_2	-0.7445151	0.623	-5.726	0.000
restaurant	Q4_3	0.1324125	0.000	NA	NA
	Q5_3	-0.6932021	1.429	-3.627	0.000
	Q6_3	-0.8062649	1.648	-3.644	0.000
	Q7_3	-0.8886584	1.807	-3.652	0.000
	Q8_3	-0.7534906	1.546	-3.637	0.000
church	Q4_4	0.4988285	0.000	NA	NA
	Q5_4	-0.7233351	0.103	-13.961	0.000
	Q6_4	-0.8767461	0.115	-15.165	0.000
	Q7_4	-0.9173310	0.118	-15.397	0.000
	Q8_4	-0.7941528	0.108	-14.571	0.000
shop	Q4_5	0.0841998	0.000	NA	NA
	Q5_5	-0.5461495	2.923	-2.215	0.027
	Q6_5	-0.7201887	3.830	-2.226	0.026
	Q7_5	-0.8648555	4.587	-2.228	0.026
	Q8_5	-0.6304172	3.362	-2.221	0.026
appointments	Q4_6	0.0891267	0.000	NA	NA
	Q5_6	-0.5864864	2.778	-2.360	0.018
	Q6_6	-0.7429692	3.496	-2.370	0.018
	Q7_6	-0.8578133	4.022	-2.373	0.018
	Q8_6	-0.6430152	3.037	-2.364	0.018
community	Q4_7	0.1365839	0.000	NA	NA
	Q5_7	-0.6395650	1.230	-3.773	0.000

(continued)

Latent Variable	Regression Variable	std.est	std.error	z	p-value
	Q6_7	-0.8631380	1.632	-3.812	0.000
	Q7_7	-0.9256781	1.743	-3.818	0.000
	Q8_7	-0.7746671	1.474	-3.801	0.000
mask	Q5_8	0.8271104	0.000	NA	NA
	Q6_8	0.9356735	0.032	35.107	0.000
	Q7_8	0.9291657	0.032	34.766	0.000
	Q8_8	0.7453217	0.036	24.765	0.000

Table S3.14: Model 3B - Trust and Risk Perception on Behavioral Intention Summary Table Output

Dependent Variables	Regression Variable	std.est	std.error	z	p-value
trust	ruralbool	-0.0507530	0.029	-1.645	0.100
	femalebool	0.0453215	0.029	1.461	0.144
	whitebool	0.1232736	0.050	3.841	0.000
	age65plusbool	0.0377615	0.031	1.215	0.224
	polVecLiberal	0.1262213	0.038	3.443	0.001
	polVecConservative	-0.4713280	0.050	-9.791	0.000
	polVecLibertarian	-0.2697549	0.091	-7.340	0.000
	polVecNonpolitical	-0.1250477	0.062	-3.656	0.000
	polVecOther	-0.0292698	0.107	-0.938	0.348
health_risk	ruralbool	-0.0129635	0.054	-0.410	0.682
	femalebool	0.1336858	0.054	4.180	0.000
	whitebool	-0.0508124	0.089	-1.588	0.112
	age65plusbool	0.2272247	0.058	7.029	0.000
	polVecLiberal	0.1561511	0.068	4.229	0.000
	polVecConservative	-0.3315642	0.070	-8.796	0.000
	polVecLibertarian	-0.2202329	0.146	-6.637	0.000
	polVecNonpolitical	-0.0451669	0.109	-1.321	0.187
	polVecOther	0.0290602	0.195	0.904	0.366
econ_risk	ruralbool	-0.0789920	0.029	-2.010	0.044
	femalebool	0.0912450	0.029	2.290	0.022
	whitebool	-0.0351966	0.047	-0.903	0.367
	age65plusbool	0.0086715	0.030	0.223	0.823
	polVecLiberal	0.0772715	0.037	1.703	0.089
	polVecConservative	-0.2524772	0.042	-4.845	0.000
	polVecLibertarian	0.0444537	0.078	1.107	0.268
	polVecNonpolitical	-0.0379610	0.059	-0.910	0.363
	polVecOther	0.0131611	0.104	0.337	0.736
beh_int	econ_risk	0.0680950	0.006	1.747	0.081
	health_risk	-0.6193686	0.017	-2.559	0.010
	trust	-0.2577844	0.013	-2.428	0.015
	ruralbool	0.0220278	0.003	0.862	0.389
	femalebool	0.0633930	0.004	1.850	0.064
	whitebool	0.0039178	0.005	0.164	0.870
	age65plusbool	-0.0178966	0.003	-0.704	0.481
	polVecLiberal	-0.0518845	0.005	-1.494	0.135
	polVecConservative	0.1485000	0.009	2.233	0.026
	polVecLibertarian	0.0194691	0.008	0.709	0.478
	polVecNonpolitical	0.0041008	0.006	0.161	0.872
	polVecOther	0.0034422	0.010	0.146	0.884
	casetodateper100	0.1044174	0.002	2.205	0.027

Note:
chisq = 10509.767 aic = 90079.661 rmsea = 0.072

Figure S3.12: Model 3B - Trust and Risk Perception on Behavioral Intention Full Diagram

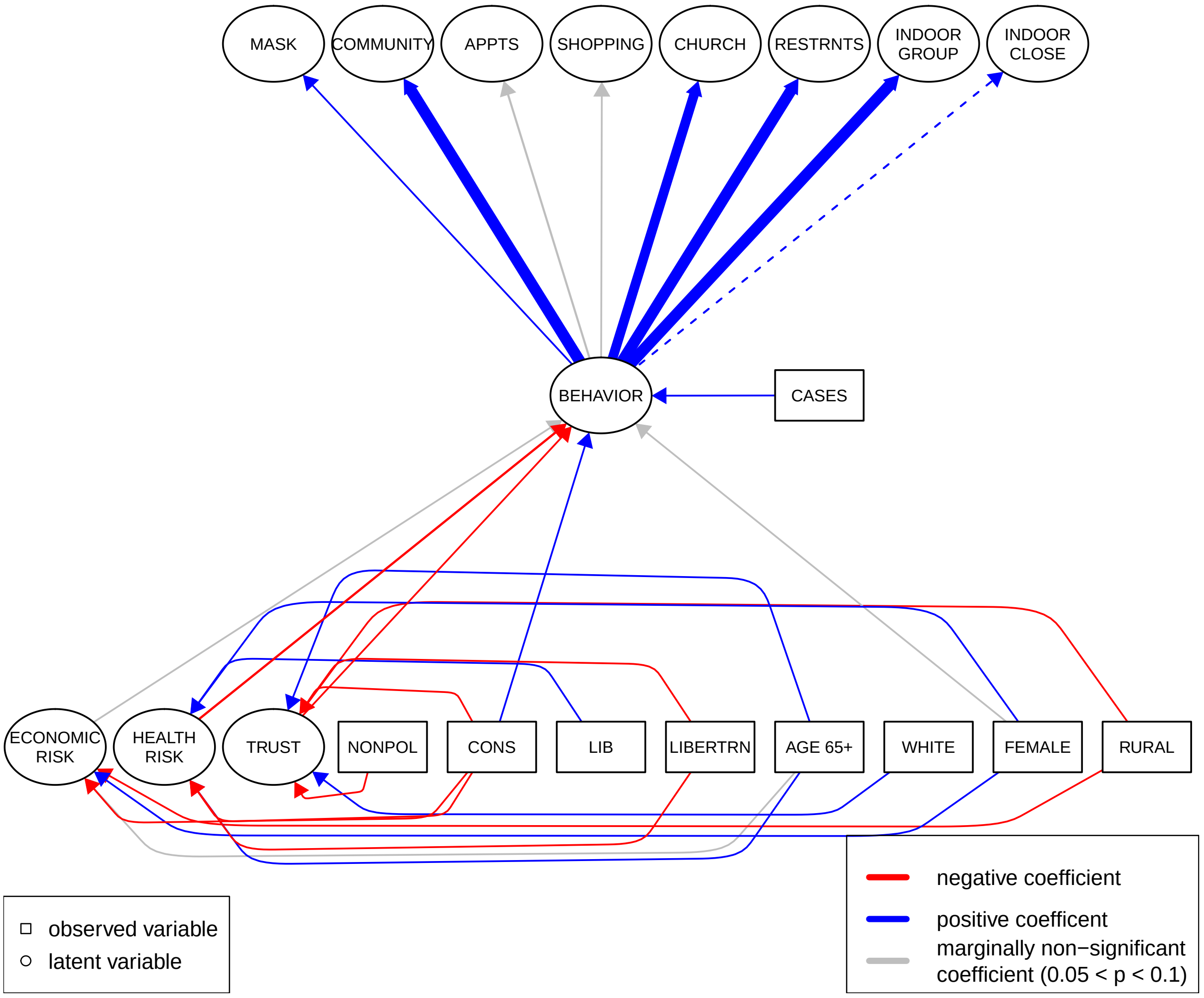

Figure S3.13: Model 3B - Trust and Risk Perception on Behavioral Intention Simplified Diagram

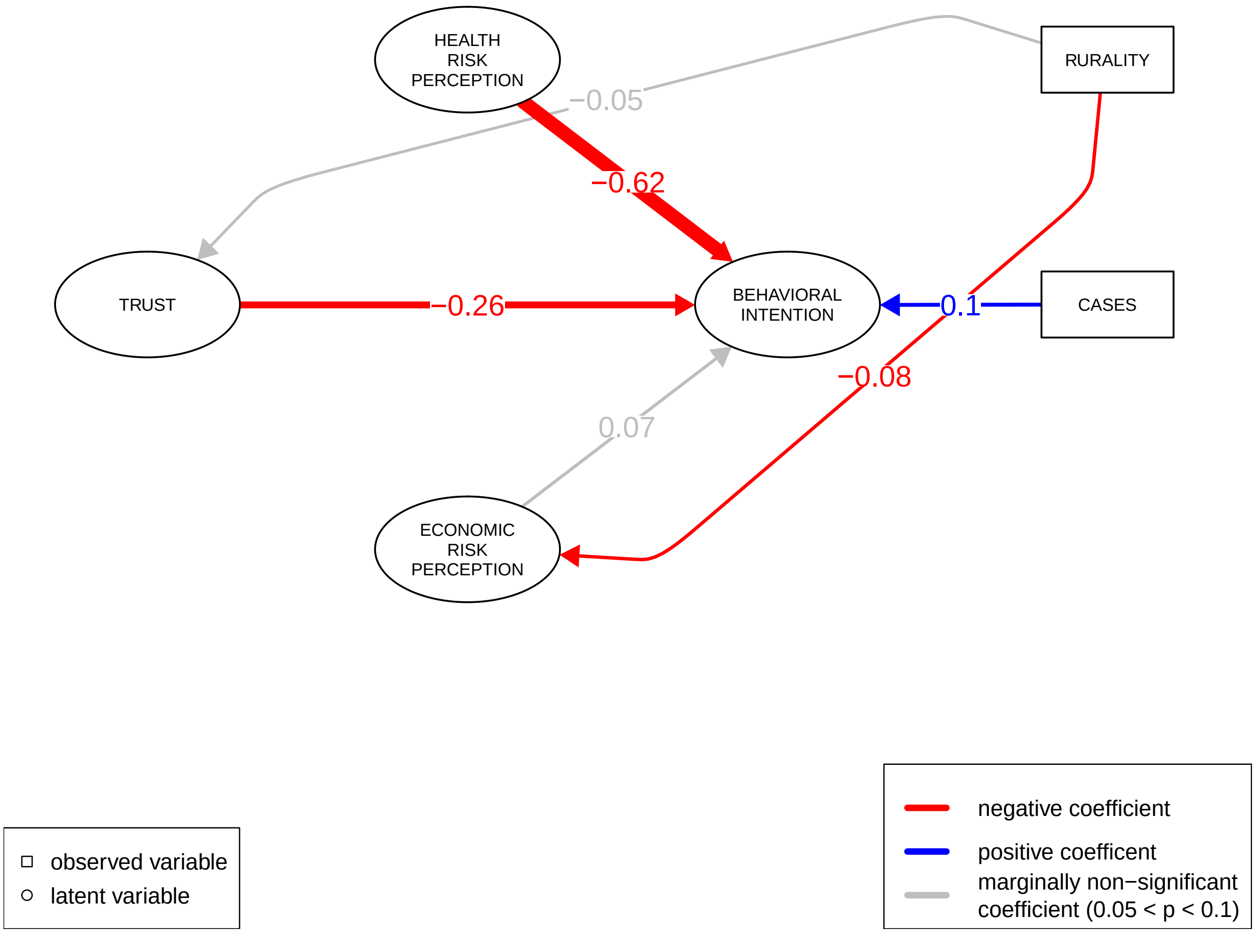

Table S3.15: Comparing Model Set A

```
## Chi-Squared Difference Test
##
##           Df   AIC   BIC Chisq Chisq diff Df diff Pr(>Chisq)
## model1A 2003 98774 99543 10470
## model2A 2004 98950 99715 10648     178.41       1  < 2.2e-16 ***
## ---
## Signif. codes:  0 '***' 0.001 '**' 0.01 '*' 0.05 '.' 0.1 ' ' 1
```

```
## Chi-Squared Difference Test
##
##           Df   AIC   BIC Chisq Chisq diff Df diff Pr(>Chisq)
## model1A 2003 98774 99543 10470
## model3A 2004 98932 99697 10630     159.91       1  < 2.2e-16 ***
## ---
## Signif. codes:  0 '***' 0.001 '**' 0.01 '*' 0.05 '.' 0.1 ' ' 1
```

Table S3.16: Comparing Model Set B

```
## Chi-Squared Difference Test
##
##           Df   AIC   BIC Chisq Chisq diff Df diff Pr(>Chisq)
## model1B 1995 89908 90668 10336
## model2B 1996 90122 90877 10552     216.12       1  < 2.2e-16 ***
## ---
## Signif. codes:  0 '***' 0.001 '**' 0.01 '*' 0.05 '.' 0.1 ' ' 1
```

```
## Chi-Squared Difference Test
##
##           Df   AIC   BIC Chisq Chisq diff Df diff Pr(>Chisq)
## model1B 1995 89908 90668 10336
## model3B 1996 90080 90835 10510     173.51       1  < 2.2e-16 ***
## ---
## Signif. codes:  0 '***' 0.001 '**' 0.01 '*' 0.05 '.' 0.1 ' ' 1
```
