# Supplementary material for: Effects of trust, risk perception, and health behavior on COVID-19 disease burden: Evidence from a multi-state US survey
Source: PLoS One. 2022 May 20;17(5):e0268302. doi: 10.1371/journal.pone.0268302 (PMC9122183; doi:10.1371/journal.pone.0268302)
Supplement: Attachment — Submitted filename: Response Letter-2-PLOS One .docx [file pone.0268302.s004.docx]

Dear editor(s),

We would like to thank the reviewer for their further helpful comments on our manuscript. In italic below, we give our responses to the comment provided by the reviewer. We have attempted to clarify/respond to comments regarding our methods with respect to the “observational unit.” We hope that this work is now acceptable for publication based on our explanation below and the minor changes to the manuscript. Thank you again for the consideration of our work for publication in PLOS ONE.

Best,

Ben Ridenhour

*Editorial Requests:*

*None.*

Reviewer #1: Referee report for “Effects of Trust, Risk Perception, and Health Behaviour on Covid-19 Disease Burden: Evidence from a Multi-State US Survey

Summary:

The responses to my points are mostly fine. With regard to my first point, the response of the authors is a little unfortunate; The Im and Kim paper that they refer to clearly uses aggregate variable as both dependent and independent variables. More precisely Im and Kim state "These 77 cities and counties were used as study units for the regression models." **Accordingly I should be more precise in my question about the paper at hand: Is the unit of observation the location or the individual?** If it's the location then you have very few data points. If it's the individual then I'm still not fully convinced by having an aggregate variable as the dependent variable.

**On the other hand, the extended answer the authors give to my point is acceptable. But I would like to see a clear statement of the unit of observation.**

*RESPONSE: We are happy that the reviewer found our extended answer in the prior revision acceptable. Here we provide a bit more detail about how the modeling works regarding the observations utilized. Unfortunately, the answer to this concern is somewhat complicated; however, we would argue, by in large and in brief, that the unit of observation for nearly all of the work done in the paper is the individual-level, and not at the county-level. The one exception to this is for the 3 sub-models of Models 1A, 2A, and 3A where a structural regression is performed with disease burden (cumulative cases per 100) as the y-variable. (Note this does not apply to any of the Models 1B, 2B, and 3B where disease burden is based on the timing of the individual response and is therefore a unique measure at the individual-level.)*

*A more in-depth, and much less brief, explanation to this is that SEM breaks down into numerous sub-models (for the lavaan package in R these are either structural regressions or confirmatory factor analyses). For example, if you look at tables S3.3 and S3.4 in the appendix, you see the results of the confirmatory factor analyses and structural regressions for Model 1A. In S3.4 with the structural regressions, the unit of observation is the individual for 4 of the 5 sub-models; the exception is the sub-model where cumcasesper100 ~ beh_int + ruralbool (indicating that disease burden is the y-variable). For all the confirmatory factor analyses—the analyses where our latent variables such as “trust” are determined by survey questions (Q26_1–Q26_8)—in Model 1A (Table S3.3) the observational unit is the individual. For reference, here is the raw summary given by lavaan of the fitted models (collectively the SEM) for Model 1A:*

```
Latent Variables:
                   Estimate  Std.Err  z-value  P(>|z|)
  trust =~
    Q26_1             1.000
    Q26_2             1.581    0.133   11.896    0.000
    Q26_3             1.221    0.115   10.579    0.000
    Q26_5             1.448    0.126   11.474    0.000
    Q26_6             1.753    0.142   12.352    0.000
    Q26_7             1.897    0.150   12.661    0.000
    Q26_8             1.804    0.145   12.467    0.000
  health_risk =~
    Q2_1              1.000
    Q2_2              0.991    0.037   26.906    0.000
    Q2_3              0.911    0.038   23.981    0.000
    Q3_1              0.959    0.037   25.697    0.000
    Q3_5              0.892    0.038   23.298    0.000
    Q3_6              0.926    0.038   24.508    0.000
  econ_risk =~
    Q3_2              1.000
    Q3_3              1.428    0.167    8.539    0.000
    Q3_4              2.093    0.233    8.978    0.000
    Q3_7              1.477    0.171    8.645    0.000
  indoor_close =~
    Q4_1              1.000
    Q5_1             -8.168    3.108   -2.628    0.009
    Q6_1             -9.054    3.441   -2.631    0.009
    Q7_1             -8.626    3.280   -2.630    0.009
    Q8_1             -5.315    2.043   -2.602    0.009
  indoor_group =~
    Q4_2              1.000
    Q5_2             -2.962    0.512   -5.785    0.000
    Q6_2             -4.185    0.703   -5.950    0.000
    Q7_2             -4.313    0.724   -5.959    0.000
    Q8_2             -3.563    0.605   -5.886    0.000
  restaurant =~
    Q4_3              1.000
    Q5_3             -5.197    1.397   -3.719    0.000
    Q6_3             -6.025    1.612   -3.737    0.000
    Q7_3             -6.621    1.768   -3.745    0.000
    Q8_3             -5.636    1.511   -3.730    0.000
  church =~
    Q4_4              1.000
    Q5_4             -1.441    0.101  -14.328    0.000
    Q6_4             -1.739    0.112  -15.571    0.000
    Q7_4             -1.815    0.115  -15.806    0.000
    Q8_4             -1.577    0.105  -14.948    0.000
  shop =~
    Q4_5              1.000
    Q5_5             -6.470    2.894   -2.236    0.025
    Q6_5             -8.522    3.793   -2.247    0.025
    Q7_5            -10.219    4.542   -2.250    0.024
    Q8_5             -7.466    3.329   -2.243    0.025
  appointments =~
    Q4_6              1.000
    Q5_6             -6.550    2.731   -2.399    0.016
    Q6_6             -8.282    3.438   -2.409    0.016
    Q7_6             -9.540    3.955   -2.412    0.016
    Q8_6             -7.181    2.988   -2.403    0.016
  community =~
    Q4_7              1.000
    Q5_7             -4.645    1.198   -3.878    0.000
    Q6_7             -6.224    1.589   -3.918    0.000
    Q7_7             -6.653    1.696   -3.923    0.000
    Q8_7             -5.593    1.432   -3.906    0.000
  mask =~
    Q5_8              1.000
    Q6_8              1.129    0.032   35.612    0.000
    Q7_8              1.121    0.032   35.259    0.000
    Q8_8              0.902    0.036   25.111    0.000
  beh_int =~
    indoor_close      1.000
    indoor_group      2.977    1.244    2.393    0.017
    restaurant        1.914    0.893    2.144    0.032
    church            6.829    2.651    2.576    0.010
    shop              0.671    0.396    1.695    0.090
    appointments      0.940    0.531    1.769    0.077
    community         1.970    0.906    2.175    0.030
    mask              8.352    3.225    2.590    0.010

Regressions:
                   Estimate  Std.Err  z-value  P(>|z|)
  trust ~
    ruralbool  (h)   -0.047    0.027   -1.707    0.088
    femalebool (m)    0.042    0.027    1.529    0.126
    whitebool         0.175    0.047    3.747    0.000
    age65plsbl        0.033    0.029    1.153    0.249
    polVecLbrl        0.124    0.036    3.469    0.001
    plVcCnsrvt       -0.468    0.049   -9.551    0.000
    plVcLbrtrn       -0.630    0.087   -7.265    0.000
    plVcNnpltc       -0.211    0.058   -3.671    0.000
    polVecOthr       -0.089    0.099   -0.899    0.369
  health_risk ~
    trust      (a)    1.121    0.112    9.992    0.000
    ruralbool  (q)    0.029    0.046    0.630    0.529
    femalebool (n)    0.176    0.046    3.804    0.000
    whitebool        -0.332    0.078   -4.271    0.000
    age65plsbl (l)    0.355    0.050    7.098    0.000
    polVecLbrl        0.148    0.060    2.482    0.013
    plVcCnsrvt       -0.088    0.068   -1.293    0.196
    plVcLbrtrn       -0.257    0.133   -1.933    0.053
    plVcNnpltc        0.087    0.096    0.909    0.363
    polVecOthr        0.256    0.169    1.518    0.129
  econ_risk ~
    trust      (c)    0.201    0.052    3.845    0.000
    ruralbool  (j)   -0.051    0.030   -1.667    0.096
    femalebool        0.064    0.031    2.105    0.035
    whitebool        -0.082    0.051   -1.617    0.106
    age65plsbl        0.003    0.032    0.095    0.924
    polVecLbrl        0.043    0.039    1.109    0.267
    plVcCnsrvt       -0.128    0.046   -2.796    0.005
    plVcLbrtrn        0.201    0.088    2.276    0.023
    plVcNnpltc       -0.011    0.062   -0.183    0.855
    polVecOthr        0.059    0.109    0.540    0.589
  beh_int ~
    econ_risk  (e)    0.009    0.006    1.573    0.116
    health_rsk (f)   -0.052    0.020   -2.596    0.009
    ruralbool  (p)    0.001    0.003    0.268    0.788
    femalebool        0.009    0.005    2.007    0.045
    whitebool        -0.007    0.006   -1.339    0.180
    age65plsbl       -0.001    0.003   -0.386    0.699
    polVecLbrl       -0.013    0.006   -2.083    0.037
    plVcCnsrvt        0.032    0.013    2.484    0.013
    plVcLbrtrn        0.019    0.011    1.759    0.079
    plVcNnpltc        0.006    0.006    0.949    0.343
    polVecOthr        0.004    0.011    0.410    0.682
  cumcasesper100 ~
    beh_int    (g)    4.327    1.743    2.482    0.013
    ruralbool  (k)   -0.553    0.065   -8.442    0.000
```

*The top block of the output (“Latent variables: …”) shows the confirmatory factor analysis sub-models; the lower block (“Regression: …”) shows the structural regression sub-models. Only the last two parameters (in bold) in the table are being fit to a sub-model where the observational unit is the county-level. In this sub-model, there would be 117 unique counts of case burden (we had respondents from 117 different counties within ID, TX, and VT). That being said, each line of data in the sub-model for “cumcasesper100” is unique because the score for the explanatory variable “beh_int” (behavioral intention) is unique to each individual; in other words, there are the full N=829 rows of data.*

*To hopefully aid our readers in understanding the unit of observation, we have added this sentence at the end of the “Statistical Models” section of our manuscript:*

*“For all of the factor analyses and structural regressions being run, the observational unit is at the individual-level with the exception of the 3 structural regression models (one each in models 1A, 2A, and 3A) where the dependent variable is cumulative cases per 100; in these three models, 117 unique values of case burden as of 30 April 2021 were available for the 117 counties sampled and were paired according to each individual's county of residence.”*
